# Plasmonic‐Hydrogel Hybrid Biomaterials Via In Situ Seeded Growth

**DOI:** 10.1002/anie.202501854

**Published:** 2025-04-22

**Authors:** Gail A. Vinnacombe‐Willson, Manuel Núñez‐Martínez, Ada Herrero‐Ruiz, Francisco Bevilacqua, Raquel Pazos, Lara Troncoso‐Afonso, Marta Gallego‐González, Leonardo Scarabelli, Luis M. Liz‐Marzán

**Affiliations:** ^1^ CIC biomaGUNE Basque Research and Technology Alliance (BRTA) Donostia‐San Sebastián 20014 Spain; ^2^ Centro de Investigación Biomédica en Red Bioingeniería, Biomateriales y Nanomedicina (CIBER‐BBN) Donostia‐San Sebastián 20014 Spain; ^3^ Department of Applied Chemistry University of the Basque Country Donostia‐San Sebastián 20018 Spain; ^4^ Department of Chemistry and Process & Resource Engineering ETSIIT University of Cantabria Santander 39005 Spain; ^5^ Ikerbasque Bilbao 48009 Spain; ^6^ CINBIO Universidade de Vigo Vigo 36310 Spain

**Keywords:** Bottom‐up synthesis, Gold nanoparticles, Hybrid plasmonic materials, Hydrogels, In situ growth

## Abstract

The combination of hydrogels and functional plasmonic metal nanoparticles affords the development of unique hybrid systems, such as actuators, biosensors, and drug delivery systems, among others. Being typically prepared in colloidal suspension, incorporating shape‐controlled plasmonic nanoparticles on polymer substrates typically requires lengthy processes involving synthesis, washing, and self‐assembly. We report an alternative, robust in situ seed‐mediated growth method whereby either isotropic or anisotropic gold and silver nanoparticles can be prepared directly on gelatin‐based hydrogels, taking advantage of the polymer's native chemical functionalities. In‐depth characterization of gold precursor–polymer interactions enabled the rational growth of branched gold nanoparticles on biocompatible hydrogels with different physicochemical properties. In situ seeded growth circumvents traditional limitations imposed by the need for colloidal stability, thereby enabling gold nanoparticle synthesis under surfactant‐free conditions and in high ionic strength solutions, thus enhancing their suitability for applications involving live cells. This method can be expanded to create libraries of hybrid plasmonic materials with potential impact in the fabrication of functional 3D cell culture substrates, as well as biological and chemical sensors.

## Graphical abstract

Detailed chemical analysis of the interactions between Au^3+^ ions and gelatin‐based hydrogels enables the development of a robust “in situ” seed‐mediated synthesis of plasmonic gold nanoparticles, whereby gold salts are reduced directly on the surface of hydrogel substrates to form the nanostructures. These hybrid plasmonic‐hydrogel materials have potential applications in 3D cell culture, tumor/tissue models, and chemical and biological sensors.

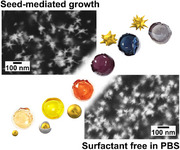

## Introduction

Plasmonic nanoparticles possess size‐ and shape‐related optical, chemical, and biological properties that facilitate their implementation as functional units in increasingly broader applications, such as environmental remediation, sensing, antifouling/antibacterial coatings, in vitro cancer and organ models, catalysis, etc. Traditionally, plasmonic gold nanoparticles have been prepared via top‐down or bottom‐up methods. While the former set of approaches (e.g., e‐beam lithography, secondary sputtering, photolithography, and nanoimprint techniques) is capable of high‐precision substrate nanopatterning, these methods usually lack compatibility with soft materials such as hydrogels due to requirements for high vacuum, high temperature, or rigid substrate supports.^[^ ^1^, ^2^, ^3^, ^4^, ^5^, ^6^ ^]^ The latter approach is the “gold standard” for controlling plasmonic nanoparticle crystal structure and morphology (and therefore high‐quality, tunable plasmonic properties).^[^ ^7^, ^8^, ^9^, ^10^, ^11^ ^]^ However, most bottom‐up syntheses generate nanoparticles in colloidal suspension, requiring various washing, functionalization, and self‐assembly steps for the nanoparticles to be anchored onto other materials or devices. In many scenarios, additional modification of the substrate material must also be carried out. Overall, complex and time‐consuming fabrication processes still represent a major bottleneck for integrating plasmonic nanoparticles into functional devices.

In this context, we aimed to further develop the technique referred to as “ in situ chemical growth,” whereby gold ions are reduced on/by a target substrate with no need for additional modifications or colloidal synthesis steps. Recent reports point toward the potential of in situ growth techniques for addressing current gaps in fabrication capabilities.^[^ ^12^ ^]^ For instance, in situ growth enabled the rapid fabrication of plasmonic nanostructures on different insulating and semiconductor oxide supports, e.g., polydimethylsiloxane (PDMS), silicon, glass, indium tin oxide, etc.^[^ ^13^, ^14^, ^15^ ^]^ In one case, the quality and position of the nanostructures were sufficiently controlled to generate a far‐field collective optical response (surface lattice resonance).^[^ ^16^ ^]^ Nanoparticles can also be directly prepared in situ on 3D structured substrates, including the internal walls of featureless and herringbone microfluidic channels,^[^ ^13^ ^]^ which are often used in biological studies.^[^ ^17^, ^18^, ^19^ ^]^ Lately, the use of in situ growth techniques for fabricating anisotropic nanoparticles on soft 3D‐printed hydrogel cell culture scaffolds has also been reported.^[^ ^14^ ^]^ These examples indicate that in situ synthesis could yield more robust, flexible, and direct fabrication routes for metamaterials, surface‐enhanced Raman scattering‐based sensors, hybrid polymeric structures for 3D in vitro models, and plasmonic‐microfluidic platforms.

Despite its considerable potential, in situ growth remains rarely exploited because the size, shape, and uniformity of in situ grown nanostructures are still poorly controlled compared to standard colloidal synthesis.^[^ ^7^, ^12^ ^]^ In this context, the biggest leap forward for colloidal synthesis toward achieving uniform shapes was the introduction of the seed‐mediated approach. In seeded growth, nucleation and overgrowth are performed sequentially so that optimal chemical environments can be applied to achieve the best results for each step. To date, most in situ synthesis methods still follow “hybrid” protocols relying on colloidal or lithographically prepared seeds,^[^ ^6^, ^13^, ^20^, ^21^ ^]^ a true adaptation of seed‐mediated growth to an in situ scheme has been reported only in a few specific examples.^[^ ^14^, ^16^, ^22^, ^23^ ^]^ For hydrogels, seeded in situ growth has not been realized, and only one‐pot seedless in situ growth has been established.^[^ ^14^ ^]^ This one‐pot approach has limitations and could be carried out on only two distinct formulations (10% w/v gelatin and gelatin methacryloyl (GelMA)). As such, the utility of in situ growth for bioengineering applications would improve significantly if comparable growth procedures could be applied on variable formulations with customizable physical, mechanical, and chemical properties. Thus, the overarching hypothesis of this work is that a detailed understanding of the chemical interactions taking place during nanoparticle growth is necessary for the development of in situ seeded growth, yielding more reliable protocols that could be translated to a wider range of polymer compositions.

To this end, we identified the need for understanding the chemical interactions between Au^3+^ salts (from HAuCl_4_) and hydrogels, which we thoroughly interrogated using Fourier transform infrared (FTIR), nuclear magnetic resonance (NMR), and ultraviolet–visible (UV–vis) spectroscopies, as well as inductively coupled plasma‐mass spectrometry (ICP‐MS). With the information gleaned from these analyses, gold nanostars (AuNSt) with high extinction in the first near‐infrared (NIR) biological transparency window (650–950 nm) were synthesized on hydrogels containing gelatin at different polymer contents (5–20% w/v), gelatin‐alginate (Gel‐Alg), and GelMA. We additionally studied the effect of gold concentration during the seeding step toward tailoring the morphology of the nanoparticle products.

Within the hypothesis that such a seeded approach would offer greater synthesis robustness, we tested its application in more challenging growth conditions. Specifically, colloidal syntheses are restricted by the need to ensure colloidal stability, which requires the addition of surfactants or other ligands. Because these molecules coat the surface of the nanoparticles, they also hinder their sensing capabilities and often produce unintended cytotoxic effects.^[^ ^24^, ^25^, ^26^ ^]^ Although a number of surfactant‐free colloidal protocols exist for AuNSt,^[^ ^24^, ^26^, ^27^, ^28^ ^]^ they usually rely on weakly bound capping ligands that stabilize the suspensions via electrostatic interactions, which are readily disrupted by ionic species.^[^ ^29^ ^]^ Using the insights gained from detailed chemical analysis, we achieved surfactant‐free in situ growth of AuNSt in high ionic strength solutions (phosphate buffered saline, PBS) as the growth media. As a result, these synthetic conditions yield plasmonic hydrogels specifically tailored for applications involving live cells.

## Results and Discussion

Gelatin and gelatin‐based hydrogels are well‐established because they are relatively easy to prepare, biocompatible, inexpensive, and commercially available, which justifies their widespread use in biodegradable packaging, extracellular matrices, substrates for cell culture, drug delivery systems, matrices for conductive and magnetic coatings, etc.^[^ ^30^, ^31^, ^32^, ^33^, ^34^ ^]^ In addition to these advantageous characteristics, pure, unmodified gelatin hydrogels in particular are convenient model systems for developing in situ seeded growth procedures because they are transparent in the 400–1100 nm range, thereby enabling optical characterization of the nanoparticle products with a standard UV–vis spectrometer. Also, aqueous gelatin solutions are liquids at 37 °C, facilitating the collection of products via centrifugation for subsequent analysis. Thus, we use pure gelatin hydrogels for our initial tests of seeded in situ growth; we subsequently explore the modification of the gels’ physical and chemical properties by tuning polymer concentration, adding other water‐soluble polymers, or introducing chemical modifications.^[^ ^33^, ^35^ ^]^

We targeted AuNSt for the development of our seeded‐growth process because they find broad applications based on their shape‐related plasmonic properties, namely, high extinction in the NIR biological transparency window,^[^ ^9^ ^]^ the presence of intense electromagnetic fields at their sharp tips (useful for sensing),^[^ ^36^ ^]^ and photothermal performance (applied in cancer therapeutics, antifouling, and heat stimulation of cells).^[^ ^37^, ^38^ ^]^ Overall, AuNSt are an ideal target shape for in situ growth because of their utility in biorelevant materials. Moreover, since AuNSt are a kinetic synthesis product,^[^ ^39^ ^]^ they can be grown relatively fast (within ∼10 min), allowing for the rapid assessment of different synthetic conditions.

**Figure 1:**
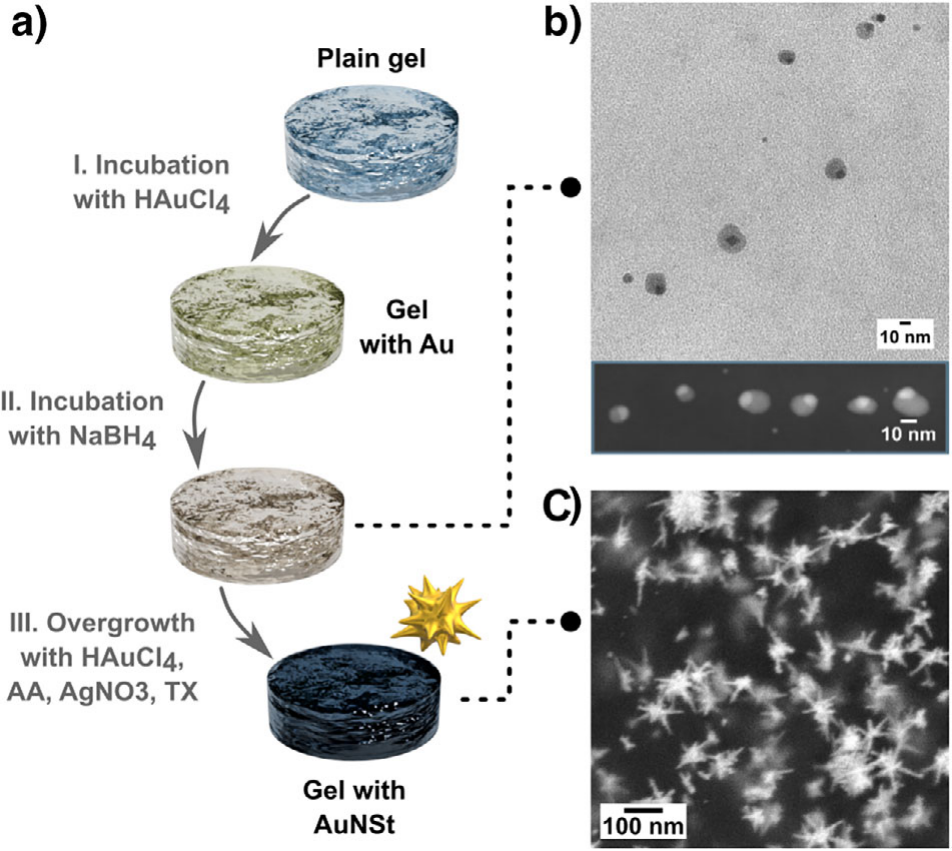
a) Scheme of the seed‐mediated growth process: (I) the hydrogel is incubated in an aqueous gold salt solution; (II) seeds are formed in situ when the gels are subsequently incubated in NaBH_4_ solution; (III) the seeds are finally overgrown into gold nanostars (AuNSt) upon incubation in a growth solution containing the shape‐directing reagents silver nitrate (AgNO_3_) and Triton X‐100 (TX), and the weak reductant ascorbic acid (AA). b) TEM image of the obtained seeds after coating them with silver so that they could be collected via centrifugation and HAADF‐STEM image confirming the chemical contrast within Ag‐coated Au seeds (inset). Control experiments and additional TEM/HAADF‐STEM characterization of the seeds are provided in Figures S1 and S2 of the Supporting Information (SI). c) Representative SEM image of the resulting AuNSt on hydrogels. Additional SEM and TEM images of the obtained AuNSt are provided in Figure S3 of the SI.

The seeded‐growth strategy was translated to hydrogels following the synthetic scheme in Figure 1a. Gelatin forms physically crosslinked hydrogels, stabilized by hydrogen‐bonding interactions, at room temperature (or below 32 °C) with no need for any other crosslinking agents.^[^ ^34^ ^]^ Accordingly, gelatin hydrogel discs were prepared by dissolving gelatin type A from porcine skin in water (10% w/v) at 40 °C for 1 h, then 200 µL aliquots were prepared in a 48‐well plate. The aliquots were finally cooled at 4 °C for 10 min until gelation was reached. Before AuNSt growth, the hydrogel substrates were warmed up to room temperature (where they were still in their gelated state). In Step I, the attachment of gold ions onto the hydrogel was performed by incubation in an aqueous solution of HAuCl_4_ under mild stirring (0.2–900 µM, 500 rpm, 5–40 min). In Step II, the gel was washed gently with MilliQ water, then introduced into a separate aqueous solution containing the strong reductant NaBH_4_, which is known to rapidly reduce Au^3+^ to Au^0^ (within <10 min), leading to the formation of gold seeds. This addition was performed under vigorous magnetic stirring (0.6 mM, 1000 rpm, 10 min), following common colloidal synthesis practices, which have shown that high stirring speeds lead to uniform seed formation.^[^ ^12^, ^16^ ^]^

Previous studies showed that the spontaneous formation of nuclei or small gold nanoparticle seeds away from the surface, so‐called “secondary nucleation,” competes with in situ growth.^[^ ^12^, ^16^ ^]^ In fact, secondary nucleation represents a major source of potential irreproducibility and nonuniformity. Therefore, the adsorption of gold ions onto the gel and the subsequent addition of NaBH_4_ were performed in separate steps to ensure nucleation only on the hydrogel substrate (Step I and II, Figure 1a). The strong reductant used in Step II (NaBH_4_) loses its reducing power over time because it readily decomposes in water. For this reason, the sample was aged for 30 min in MilliQ water after completion of Step II to avoid further nucleation. The gel was rinsed again with MilliQ water, then in the growth step (Step III), the adsorbed nuclei are overgrown. First, a separate vial containing a growth solution tailored for AuNSt was prepared containing the shape‐directing reagents silver nitrate (AgNO_3_, 60 µL, 10 mM) and hydrochloric acid (HCl, 150 µL, 1 M), the surfactant Triton X‐100 (TX, 5 mL, 100 mM), and additional gold precursor (HAuCl_4_, 60 µL, 50 mM). Subsequently, the weak reducing agent ascorbic acid (AA, 160 µL, 100 mM) was rapidly added to the growth solution under vigorous stirring (1000 rpm). Once the yellowish solution became colorless (after ∼15 s, indicating complete reduction of Au^III^ into Au^I^), the gel disc containing gold seeds was added to the AuNSt growth solution and incubated for 5 min under stirring at 1000 rpm (within this time Au^I^ is reduced to Au^0^ on the gold seeds). Finally, the gels were rinsed thoroughly with MilliQ water.

Then, the prepared seeds and AuNSt were characterized by electron microscopy (Figure 1b,c). One of the major challenges for in situ growth is that, unlike in colloidal synthesis, the small gold nanoparticle nuclei (“seeds”) are bound onto the surface of a support material. Therefore, it is difficult to characterize them by standard transmission electron microscopy (TEM). We also found that the formed nuclei were too small and/or sparse to be observed directly on the hydrogel by scanning electron microscopy (SEM). As an added complication, particles with diameters <5 nm are unstable over time and tend to coalesce.^[^ ^40^ ^]^ Their size, morphology, or crystal structure may thus be affected during the heating step required to collect them from the hydrogel.^[^ ^40^, ^41^ ^]^ Therefore, we performed a silver coating reaction after Step II to protect the seeds and increase their dimensions so they could be collected by centrifugation after dissolving the gel (18 krpm or ∼3.6 × 10^5^ g for 60 min, washed 2 to 3 times with MilliQ water). Owing to the silver coating, successful preparation of small gold seeds was confirmed by TEM (Figures 1b, S1, and S2). Compared to standard TEM, high‐angle annular dark field‐scanning transmission electron microscopy (HAADF‐STEM) is more sensitive to elemental composition because it is not susceptible to contrast reversal due to changing the focus of the electron beam.^[^ ^42^ ^]^ Therefore, HAADF‐STEM was also used to confirm the core‐shell nature of the coated seeds (Figures 1b (inset, core size ∼8 ± 4 nm), S1, and S2). We note that cores smaller than 2 nm may exist, but distinguishing the core‐shell structure and/or isolating them by centrifugation would be too difficult. The formation of seeds was further supported through a series of control experiments, demonstrating that AuNSt growth does not occur unless both Steps I and II are completed (Figure S1A).

The AuNSt growth protocol used in Step III closely follows colloidal protocols for TX‐stabilized AuNSt, with some modifications.^[^ ^9^, ^43^ ^]^ In the reported syntheses, ascorbate is a more powerful reductant compared to its protonated form, so here HCl is added to effectively slow the reaction and help inhibit secondary nucleation.^[^ ^9^, ^16^, ^43^, ^44^ ^]^ The resulting AuNSt exhibited an extinction band in the NIR range as expected, and both SEM and TEM characterization showed that the products featured spiky, branched geometries (Figures 1c and S3).  Although the primary interest of this work is the synthesis of AuNSt due to their utility for bioapplications, we also take the opportunity to perform a proof‐of‐concept demonstration regarding the versatility of in situ growth to enable the formation of different morphologies by varying the composition of the growth solution. As expected, when using a growth solution with reagents selected to favor gold nanospheres (containing only HCl, ascorbic acid, and HAuCl_4_), isotropic nanoparticles were obtained (Figure S4). In addition to growth solution composition, incubation time during Step III can be readily controlled via removal of the sample from the growth solution, which could allow for the selection of products with different sizes and plasmonic properties (Figure S5). Moreover, for Step III, stirring speeds >500 rpm proved to be required for achieving AuNSt (Figure S6). Altogether, our results support the formation of seeds on the hydrogel and their subsequent overgrowth into the selected gold nanoparticle geometry.

**Figure 2:**
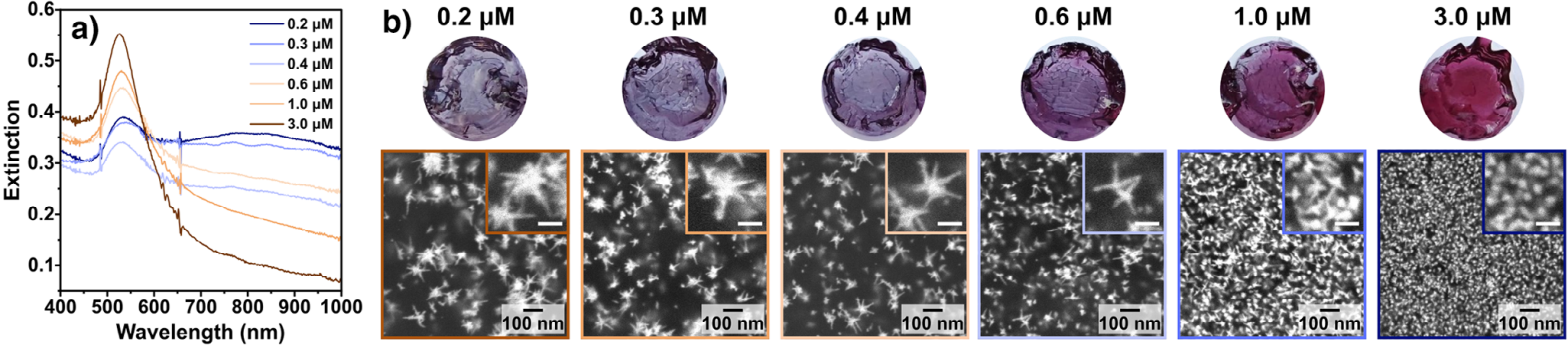
a) Extinction spectra of different products obtained by changing the concentration of gold salt during the seeding conditions on 10% w/v gelatin substrates. b) Digital photographs of the obtained hydrogels (top) and their corresponding SEM images (bottom). Size distributions, additional SEM images, and analysis of the electron microscopy images are presented in Figures S7–S9. From left to right, the nanoparticle dimensions are 81 ± 34, 66 ± 30, 58 ± 27, 50 ± 19, 42 ± 17, and 20 ± 6 nm.

Next, we demonstrate that, like in colloidal seeded growth, altering the conditions of the seeding step influences the plasmonic properties of the overgrown nanoparticles (Figure 2). By modulating the HAuCl_4_ concentration during the seeding steps, the density, size, and yield of anisotropic products can be varied (Figures S7–S9). As the gold concentration was increased during seeding (Figure 1a, Step I), the extinction at ∼530 nm increased while the intensity of the band in the NIR decreased (Figure 2a). Usually, AuNSt present two bands in the UV–vis‐NIR spectra: a low‐intensity band/shoulder around 530 nm from the plasmon mode of the AuNSt core and another more prominent and broader band at higher wavelengths corresponding to the hybridized plasmon modes of the core and branches.^[^ ^45^ ^]^ The position of the peak at higher wavelengths depends on the aspect ratio of the nanostar branches, with “sharper,” higher aspect ratio branches giving more red‐shifted signals.^[^ ^9^ ^]^ The plasmon mode of the branches can be tuned anywhere from ∼650 nm to deep into the NIR (>1100 nm), depending on the desired plasmonic properties.^[^ ^9^, ^43^, ^46^ ^]^ Isotropic products with no branching also have a plasmon resonance that contributes to the peak at ∼530 nm.^[^ ^46^ ^]^ SEM characterization showed that the observed changes in extinction correlated well with the obtained geometries (Figure 2b). Analysis of the aspect ratio of the particles grown under the different seeding conditions shown in Figure 2 revealed a slight decrease in both branch length and width along with increasing HAuCl_4_ concentration during seeding Step I (see Figure S9). However, significant changes were observed in the yield of branched particles, the number of branches, and the overall particle size, which all increased as the concentration of HAuCl_4_ was decreased during seeding Step I. Therefore, the yield of highly branched particles was also increased, causing the relative intensity of the LSPR band in the NIR to increase compared to that around ∼530 nm. A larger number of seeds on the gel would change the ratio of reactants during the growth step ([HAuCl_4_]:[seeds]) and in turn reduce the growth rate. As a result, isotropic growth would be favored, consistent with our results.

We note that, for [HAuCl_4_] ≥ 100 µM during seeding Step I, there was a visible and measurable (via UV–vis) color change from clear to brown or red, signaling the formation of small particles after both seeding steps (Figure S10). The seeds reached a size measurable by SEM (17 ± 4 nm) when [HAuCl_4_] ≥ 1.2 mM was used during seeding (60‐fold the amount needed to obtain stars with the highest NIR extinction; Figure S10). Although these conditions did not yield AuNSt, we used these data to infer that both the size and density of the seeds increase with increasing HAuCl_4_ concentration. We additionally demonstrate that the extinction maxima can be readily tuned by modifying the amount of gold during seeding, as expected for a seed‐mediated growth scheme.

**Figure 3:**
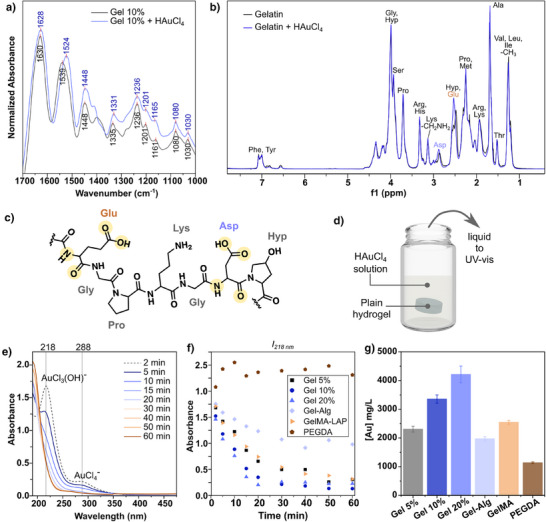
a) FTIR spectra comparing gelatin 10% w/v (“Gel 10%”) without and with incubation for 5 min in an aqueous solution of 50 mM HAuCl_4_ (“Gel 10% + HAuCl_4_”). b) ^1^H‐NMR spectra and peak assignments for gelatin/D_2_O solution with and without the addition of aqueous HAuCl_4_. Full FTIR spectra, COSY spectra, and ^1^H‐NMR peak assignments are provided in the Supporting Information (Figures S11 and S12). c) Schematic highlighting the residues where NMR peak shifts were observed (according to color) and the proposed interaction sites for gold ions (yellow highlighted). d) Schematic showing the evaluation of gold binding to hydrogels by UV–vis spectroscopy: the as‐prepared hydrogels are incubated in a 0.2 mM aqueous solution of HAuCl_4_ and the UV–vis spectrum of the gold precursor solution is measured at different time points. e) UV–vis spectra of the collected aqueous gold precursor solution for gelatin 10% w/v showing the decreasing intensity of the peaks corresponding to chlorinated and hydroxylated Au^3+^ salts over time (*l *= 0.5 cm). f) Comparison of the change in intensity of the peak at 218 nm over time for different hydrogel formulations: gelatin 5%, 10%, and 20% w/v (“Gel 5%,” “Gel 10%,” and “Gel 20%,” respectively), gelatin 10% w/v mixed with alginate 2% (“Gel‐Alg”), gelatin methacryloyl 10% w/v (“GelMA”), and poly(ethylene glycol) diacrylate 10% v/v (“PEGDA”) (full data presented in Figure S17). g) ICP‐MS quantification of gold on the hydrogels after 5 min incubation in 1 mL of 50 mM HAuCl_4_ aqueous solution.

We hypothesized that improving our understanding of the chemical interactions behind in situ growth would be key toward translating it to various types of hydrogels. In the literature, there have been a number of both computational and experimental studies detailing the interaction of Au^3+^ ions with polar (amine, amide, hydroxyl, carboxyl) groups.^[^ ^47^, ^48^, ^49^, ^50^ ^]^ We performed a series of FTIR, proton NMR (^1^H‐NMR), and correlation spectroscopy (COSY) characterizations to identify the relevant functional groups for gold–gelatin interaction (Figures 3, S11, and S12). For the FTIR study, two substrates were prepared: (1) plain 10% w/v gelatin and (2) 10% w/v gelatin incubated in an aqueous solution of 50 mM HAuCl_4_. The FTIR samples were prepared with a high concentration of gold precursor to ensure the presence of Au^3+^. However, this condition requires protection from light and rapid sample processing because amines and tertiary amides are capable of light‐catalyzed reduction of Au^3+^ into Au^I^, which would inhibit our study of gelatin–Au^3+^ interaction.^[^ ^47^ ^]^ Both samples were then frozen quickly in liquid nitrogen, wrapped in aluminum foil to protect them from light, lyophilized overnight, then analyzed by FTIR. Overall, we found that key peaks at 1630, 1540, and 1335 cm^−1^ assigned to the amide I, II, and III regions (attributed mainly to C═O stretching, N─H bending, and C─N stretching, respectively, Figure 3a) shift to lower frequencies.^[^ ^51^, ^52^, ^53^ ^]^ These shifts indicate an interaction between gold ions and amide groups, in agreement with previously reported results based on Raman spectroscopy.^[^ ^14^ ^]^

Subsequently, we used NMR to probe more specifically which residues play major roles in gold binding. Gelatin from porcine skin has a high content of glycine (Gly; ∼185 g kg^−1^), proline (∼120 g kg^−1^), glutamic acid (Glu; 97 g kg^−1^), hydroxyproline (93 g kg^−1^), arginine (82 g kg^−1^), alanine (73 g kg^−1^), and aspartic acid (Asp; 64 g kg^−1^), with all other amino acids making up less than 50 g kg^−1^ each.^[^ ^54^ ^]^ 1D and 2D ^1^H‐NMR spectra were obtained for 1 mg mL^−1^ gelatin in D_2_O solution, both with and without addition of 4 µL mL^−1^ of 50 mM aqueous HAuCl_4_ (Figures 2b and S11).

COSY analyses along with literature reports were used to assign the peaks to specific amino acids.^[^ ^55^, ^56^ ^]^ Both Glu and Asp contain carboxylic acid groups and exhibit shifts in their corresponding CH_2_ peaks. Interestingly, no shifts were found in peaks corresponding to the CH_2_ groups of lysine (Lys), indicating negligible interaction between gold and the primary amine group.

Additionally, no shift was observed in the CH_2_ group of Gly, suggesting that the secondary amides alone may not be the most attractive groups for gold binding. Although previous density functional theory (DFT) studies indicate that the amide terminal of proline groups can interact strongly with gold, we did not observe any obvious shifts.^[^ ^57^ ^]^ Further, gold salts have a tendency to interact or chelate with hydroxyl and carboxyl groups.^[^ ^58^, ^59^, ^60^ ^]^ Considering this background along with the NMR results, which indicate interactions at polar amino acid residues, and the FTIR characterization, which indicates gold binding at amide moieties, we propose that gold ions bind to the gels via chelation to the polar Glu and Asp residues and their neighboring amides, similar to recent reports on other peptide‐based materials (Figure 3c).^[^ ^50^ ^]^

Equipped with knowledge regarding gold–gelatin interactions, we next assessed the gold ion–hydrogel interactions for a variety of hydrogel formulations, applying different polymer contents and compositions that emulate formulations frequently used in 3D cell culture models^[^ ^61^, ^62^, ^63^, ^64^, ^65^, ^66^ ^]^ and biosensing platforms,^[^ ^67^, ^68^ ^]^ with the goal of expanding the range of materials on which in situ AuNSt growth could be performed.  Namely, we used both UV–vis spectroscopy and ICP‐MS to evaluate gold sequestration by hydrogels composed of gelatin at different fractions (from 5% to 20% w/v), a mixture of 10% w/v gelatin with 2% w/v alginate (Gel‐Alg), gelatin methacryloyl (GelMA, 10% w/v), and poly(ethyleneglycol)diacrylate (PEGDA 10% w/v). These materials provide a range of rheological and mechanical properties (rheological and swelling characterization is summarized in Figures S13–S15 in the Supporting Information). For UV–vis experiments, the plain hydrogels were incubated in an aqueous solution of HAuCl_4_ (0.2 mM) under mild stirring (250 rpm), and the absorbance of the solution was measured at different time points to monitor the concentration of gold salt remaining in solution (Figures 3d–f and S16). The HAuCl_4_ solution contains AuCl_4_^−^ as well as other hydroxylated species (e.g., AuCl_3_(OH)^−^), which have ligand‐metal transition bands at ∼218 and ∼288 nm (Figure 3e).^[^ ^69^ ^]^ Monitoring the decrease in intensity for the peak at ∼218 nm resulted in a trend in gold sequestration from most to least absorbed gold as: gelatin 20% w/v, gelatin 10% w/v, GelMA 10% w/v, gelatin 5% w/v, Gel‐Alg 10% and 2% w/v, and PEGDA 10% v/v (Figure 3f, see Figure S16 for details and control experiments). Complementary ICP‐MS characterization showed the same trend (Figure 3g).

Taking the information from the above spectroscopic characterization into consideration, we addressed next in situ seeded growth on the different gel types. In the case of PEGDA, only ester groups are present, thus minimal interactions with gold ions being observed by UV–vis spectroscopy and ICP‐MS (Figure 3f,g). Additionally, FTIR and NMR spectra gave no shifts upon addition of gold precursors to PEGDA hydrogels. Indeed, when AuNSt growth was tested on PEGDA, few branched products formed, and thus no additional optimization was pursued (Figure S17).

**Figure 4:**
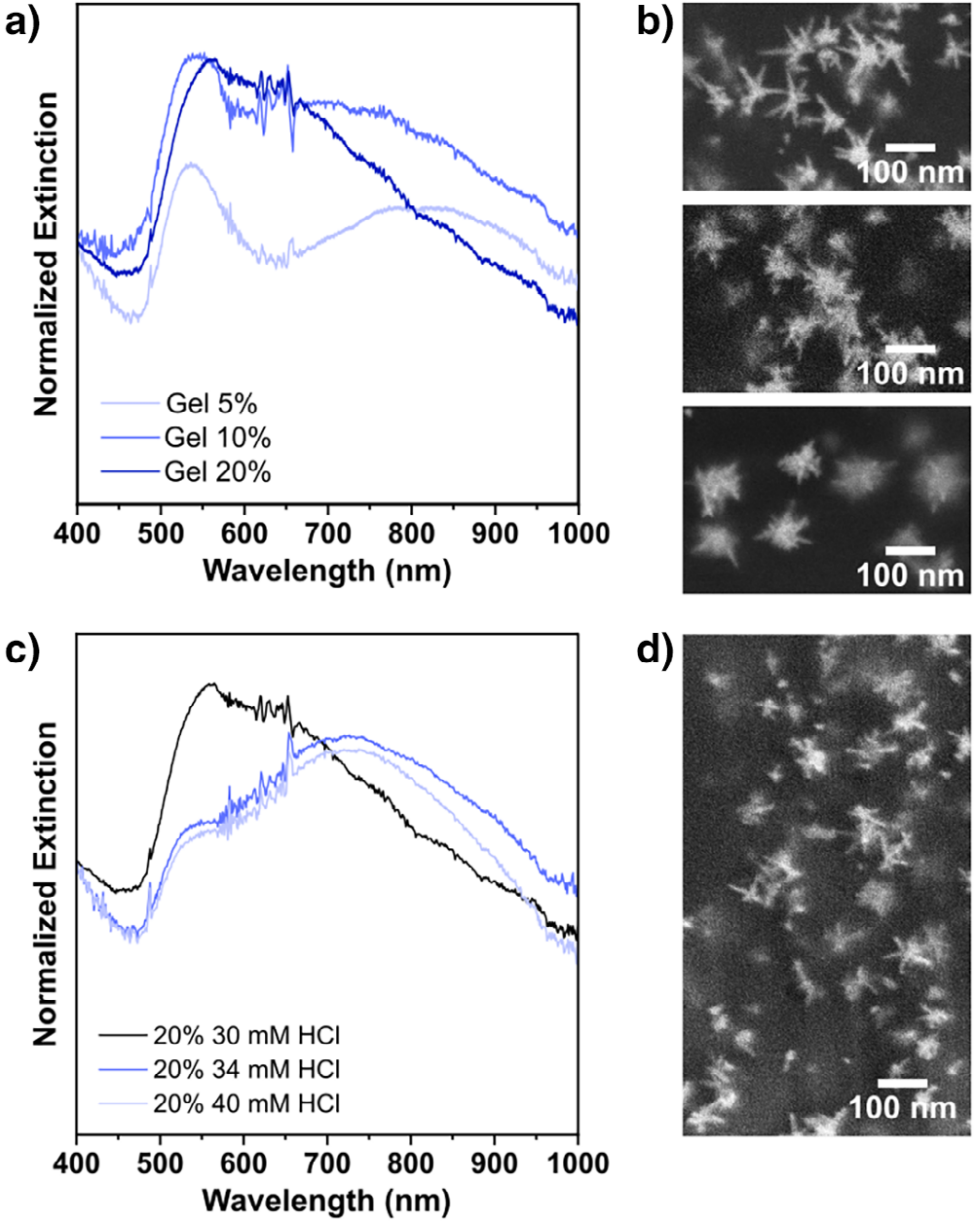
a) Extinction spectra normalized at 400 nm to comparin the position of the extinction maxima of the products obtained under the same growth conditions for 5, 10, and 20% w/v gelatin hydrogels. b) The corresponding SEM images: 5% (top), 10% (middle), 20% (bottom). c) UV–vis spectra comparing the normalized extinction of AuNSt grown on 20% w/v gelatin hydrogels through HCl addition (HCl concentration ramps for 5% and 10% are shown in Figure S19). Spectra were normalized at 400 nm to facilitate comparison of the peak intensities at the extinction maxima. d) SEM image of an optimized sample.

We next focused on the in situ seeded AuNSt growth on gelatin substrates at different polymer concentrations (Figure 4a,b). Based on spectroscopy and ICP‐MS studies, a higher percentage of gelatin results in increased gold binding and, as a result, higher extinction around ∼530 nm after the growth step. Since amines and amides have the capability to fully reduce Au^3+^ into Au^0^ in the presence of light,^[^ ^47^, ^70^ ^]^ we hypothesized that the increased presence of amides might contribute to the production of isotropic byproducts via secondary nucleation. In fact, when the 10% w/v gelatin hydrogel was dissolved following AuNSt growth, a population of isotropic particles was also collected, which contribute to the absorbance at ∼530 nm (Figure S18). Consequently, we hypothesized that slowing the reaction using HCl would offset the higher presence of amides and other polar groups (as described above and previously reported^[^ ^16^ ^]^). As predicted, the increase in HCl concentration reduced the intensity of the absorbance associated with the byproducts (Figure 4c,d). Concentration ramps of HCl were also performed for 5% and 10% w/v gelatin (Figure S19). In line with our hypothesis, higher HCl concentrations were required to decrease the presence of secondary nucleation byproducts as gelatin concentration was increased.

**Figure 5:**
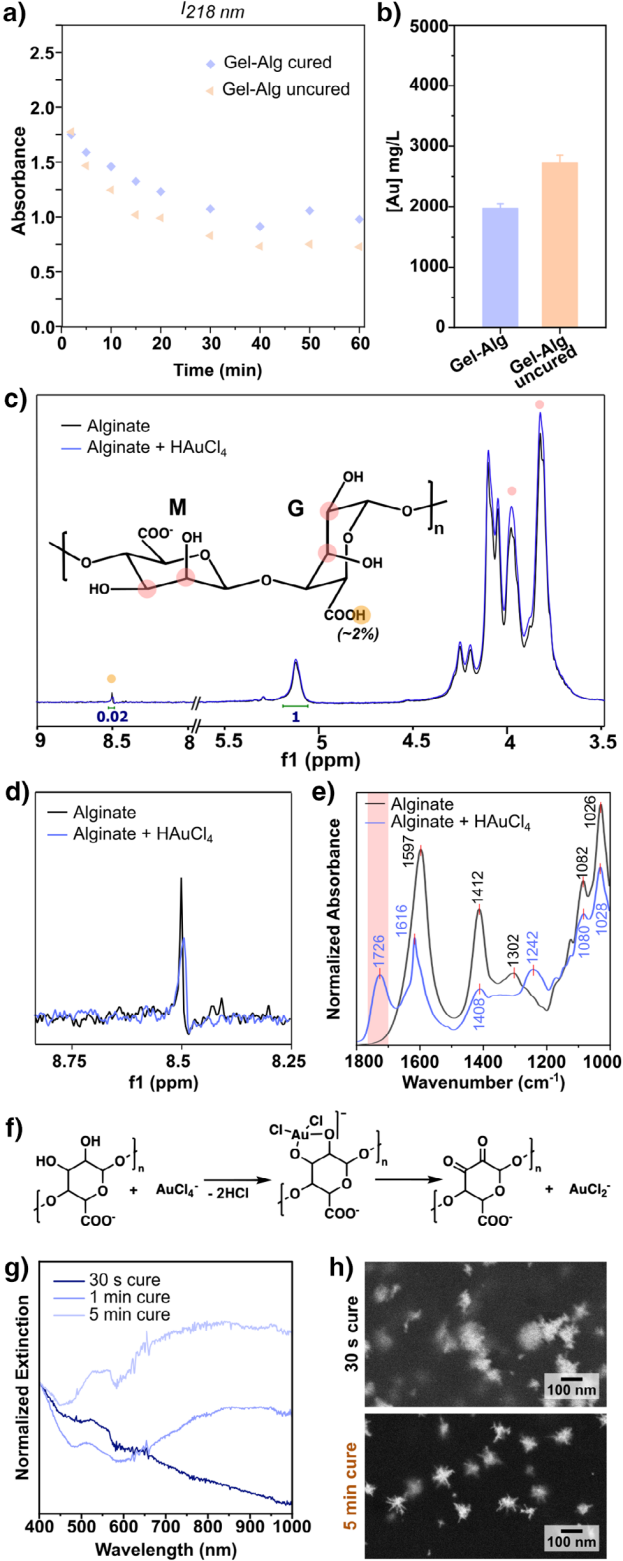
a) Comparison of gold ion sequestration from solution for cured and uncured Gel‐Alg, determined by UV–vis spectroscopy (compared to UV–vis and ICP‐MS data on cured Gel‐Alg from Figure 3f,g; *l *= 0.5 cm; full data shown in Figure S16). b) Quantity of gold bound to uncured and cured Gel‐Alg after 5 min of incubation in 50 mM HAuCl_4_, determined by ICP‐MS (plotted at the same scale as Figure 3g for comparison). c) ^1^H‐NMR spectra comparing alginate in D_2_O, with and without the addition of HAuCl_4_ (full ^1^H‐NMR data shown in Figure S11) and chemical structure with the identified shifted peaks highlighted (red = ─CH─OH groups and orange = ─COOH). d) Zoom‐in of the ^1^H‐NMR spectra. e) FTIR spectra of freeze‐dried alginate gels without incubation and with 5 min incubation in 50 mM HAuCl_4_ (FTIR spectra of Gel‐Alg with and without gold shown in Figure S12). f) Reduction mechanism of Au^III^ into Au^I^ by alginate. g) Normalized extinction spectra of overgrown products obtained on Gel‐Alg substrates, prepared with different curing times. Spectra were normalized at 400 nm to enable comparison of the peak intensities at the extinction maxima. h) SEM images of the morphology of the products obtained with 30 s and 5 min curing times. Additional SEM images are provided in Figure S22.

We then analyzed a case where gelatin was mixed with alginate, a natural polymer extracted from kelp or brown algae. Alginate is often added to gelatin or GelMA to enhance the hydrogel's stability at higher temperatures or its rigidity for cell culture applications.^[^ ^33^, ^67^ ^]^ In alginate hydrogels, the hydroxyl and carboxyl groups of the polymer form ionic complexes with M^2+^ ions, in contrast to thermally crosslinked pure gelatin hydrogels,^[^ ^71^ ^]^ usually employing Ca^2+^ salts as gelling agents. Similar to gelatin, ICP‐MS and UV–vis were used to compare the interactions of both uncured and cured Gel‐Alg with gold ions (Figure 5a,b). Non‐crosslinked Gel‐Alg sequestered more gold than ionically crosslinked Gel‐Alg, indicating that carboxyl and hydroxyl groups in alginate can cause additional gold binding or undesired side reactions.^[^ ^72^, ^73^, ^74^, ^75^ ^]^ Moreover, Gel‐Alg substrates cured in CaCl_2_ solutions at incubation times ≥5 min all yielded similar products (Figure S20).

The gold‐alginate interaction was further interrogated by ^1^H‐NMR and FTIR spectroscopies. The recorded ^1^H‐NMR spectra exhibited shifts in both the carboxyl and hydroxyl groups upon introduction of gold salts to an alginate solution in D_2_O, indicating gold‐alginate interaction at those moieties (Figure 5c,d). Alginate consists of blocks of guluronic acid (G) and mannuronic acid (M), which are not necessarily present in the same proportion and can vary depending on the source. Moreover, alginate exists primarily in its ionized form (COO⁻), although a minor fraction remains as carboxylic acid (COOH), as indicated by the weak signal observed at 8.53 ppm in the NMR spectrum (around 2%, Figure 5c,d). FTIR spectra of Gel‐Alg after gold salt incubation led to changes in the amide regions (like plain gelatin), but also a new shoulder around 1726 cm^−1^, indicating the formation of carbonyl groups (Figure S12). In the plain alginate gel, the new peak at 1726 cm^−1^ was even more prominent upon the introduction of gold precursors (Figure 5e). Following previous reports consistent with our spectroscopic analyses, we propose that the reduction of AuCl_4_^−^ to AuCl_2_^−^ by alginate occurs following the mechanism in Figure 5f.^[^ ^72^, ^73^, ^74^, ^75^ ^]^ It is well known that AuCl_2_^−^ can undergo a disproportionation reaction (3AuCl_2_^−^ (aq) → AuCl_4_^−^ (aq) + 2Au^0^ (s) + 2Cl^−^ (aq)), leading to the formation of gold nanoparticles outside of our intended growth scheme.

As mentioned above, the carboxyl and hydroxyl groups of alginate participate in the ionic crosslinking with M^2+^ ions, forming so‐called “egg‐box” dimers (Figure S21).^[^ ^71^ ^]^ This offers an explanation as to the difference between gold binding in cured and uncured Gel‐Alg. In well‐cured hydrogels, the moieties that cause unwanted side reactions are occupied in forming ionic bonds. As a result, proper gelation of alginate is crucial for controlled seed‐mediated AuNSt growth. Predictably, samples gelated for longer times were more appropriate for the growth of branched AuNSt. As shown in Figure 5g, the NIR extinction corresponding to the branched products increased significantly with the curing time (i.e., incubation time in 100 mM CaCl_2_). The SEM characterization in Figure 5h confirms the presence of branched products on fully cured hydrogels (additional SEM images are provided in the SI Figure S22).

Next, in situ seeded AuNSt growth was optimized on a covalently crosslinked hydrogel, GelMA. The amine and hydroxyl residues of lysine and tyrosine, respectively, in gelatin are modified with methacrylic anhydride to create GelMA.^[^ ^35^ ^]^ The typical yield of the methacrylation was ∼80% as evaluated by ^1^H‐NMR (described in the SI, see Methods, Section D, Figure S23). GelMA crosslinking relies on the addition of a photoinitiator to create covalent bonds between the methacrylate groups. In situ growth was tested on two 10% w/v GelMA formulations with 5% and 1.5% w/v (2‐hydroxy‐4′‐(2‐hydroxyethoxy)‐2‐methylpropiophenone (irgacure, Ig) or lithium phenyl (2,4,6‐trimethylbenzoyl) phosphinate (LAP), respectively, as photoinitiators, following previously reported preparation methods.^[^ ^76^ ^]^ The choice of photoinitiator often depends on the desired application. For instance, GelMA hydrogels prepared with LAP provide better biocompatibility, lower degradation rates, smaller pore sizes, and a lower degree of swelling.^[^ ^76^ ^]^ Overall, the addition of methacrylate groups decreases the charge compared to plain gelatin due to the functionalization of primary amines. Moreover, GelMA is more rigid/swells less than gelatin and is more stable against degradation due to its covalent bonds (Figures S13–S15).

**Figure 6:**
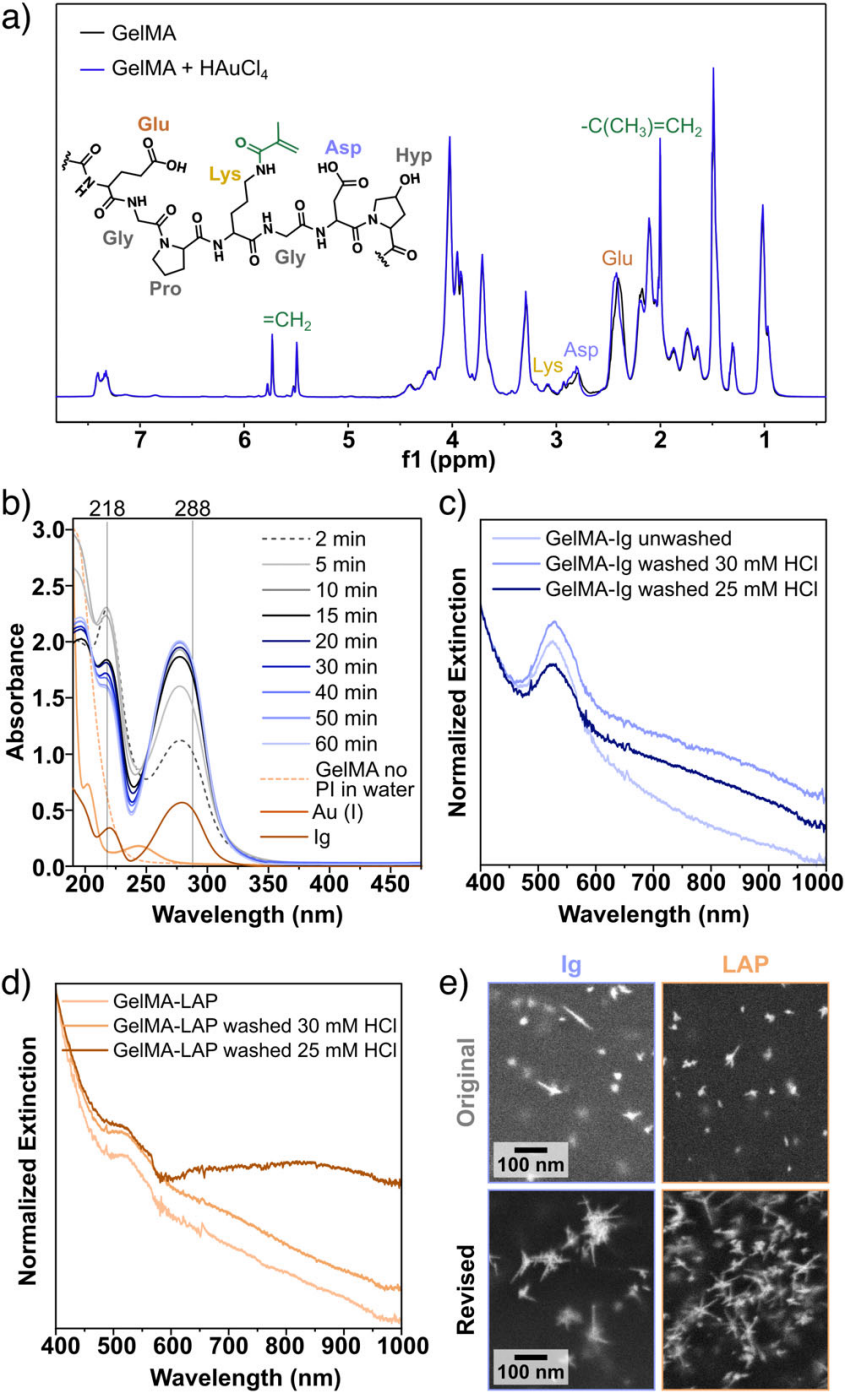
a) ^1^H‐NMR spectra of a 10 mg mL^−1^ D_2_O solution of GelMA, before and after adding 4 µL mL^−1^ of 50 mM HAuCl_4_. The different R groups and positions on the methacrylate group are highlighted according to a color code (full ^1^H‐NMR data shown in Figure S11 ) UV–vis spectral traces during a gold binding experiment for GelMA‐Ig (*l *= 0.5 cm). c) and d) Normalized extinction spectra illustrating the optimization of AuNSt synthesis on GelMA‐Ig c) and GelMA‐LAP d). Spectra were normalized at 400 nm to enable comparison of the peak intensities at the extinction maxima. e) SEM images of the products obtained before and after optimization. Additional SEM images are provided in Figure S24.

Spectroscopic analysis was again used to assess which functional groups were important for gold ion binding. FTIR and ^1^H‐NMR characterization did not indicate gold binding to methacrylate groups (Figures 6a and S12). On the other hand, excess photoinitiator was found to leach into solution and cause the unwanted reduction of Au^III^ into Au^0^ during the UV–vis monitored binding experiment (Figure 6b). Actually, both Ig and LAP contributed to this side reaction. Indeed, washing the hydrogels in MilliQ water overnight to remove excess photoinitiator led to an improvement in the degree of branching of AuNSt synthesized on these gels (Figures 6c–e and S24). Even after washing the samples, the growth of highly branched structures was more inhibited for GelMA compared to gelatin. We hypothesized that this result may be due to various factors: (i) stronger Coulombic repulsion between the gel and AuCl_4_^−^, (ii) lower swelling of GelMA in the growth solution, or (iii) the presence of remaining photoinitiator causing secondary nucleation. While we were unable to pin down the exact contributions behind the lower yield of AuNSt, we addressed it by decreasing [HCl] to promote kinetic growth (Figures 6c–e and S24C,F).

So far, we have established a seeded growth method to prepare branched nanoparticles, which does not require colloidal synthesis steps and can be translated to a variety of hydrogel formulations. In fact, seed‐mediated in situ growth is robust enough that it provides opportunities for realizing the growth of AuNSt under “unconventional” synthetic conditions. To demonstrate this, we used in situ seeded growth to prepare AuNSt in high ionic strength media without adding any surfactants for the preparation of biocompatible plasmonic materials.

**Figure 7:**
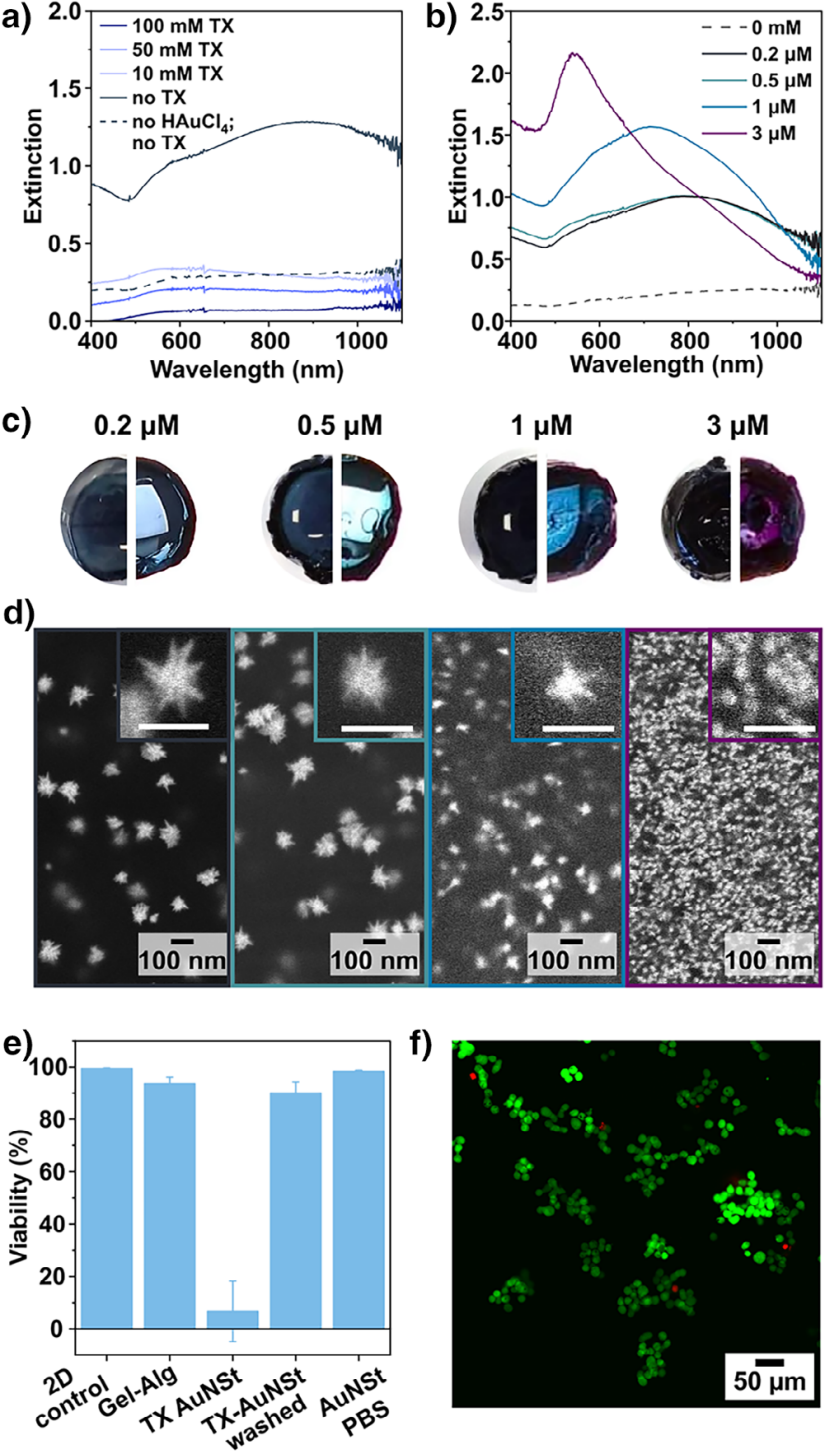
UV–vis spectra for AuNSt grown in PBS with a) different concentrations of Triton X‐100 (TX) as a capping ligand and b) different concentrations of gold salt during the seeding step. c) Digital photographs of the hydrogels prepared for the different seeding conditions on a white background (left) and in front of a light source (right). d) SEM images of the corresponding products observed on the surface of the gel. Average sizes measured from *n* = 150 nanoparticles were 80 ± 23, 79 ± 30, 36 ± 13, and 21 ± 6 nm, from left to right (Figure S26). e) Comparison of 24 h viability of seeded MDA‐MB‐231 cells on Gel‐Alg with *in* *situ* AuNSt prepared under different conditions. f) Fluorescence microscopy live/dead staining with calcein (green; live) and propidium iodide (PI, red; dead) evaluating cell viability after 24 h on the gels in vitro. Additional electron microscopy images of the products formed on gelatin and gelatin‐alginate, as well as fluorescence microscopy images, are provided in Figures S25–S31.

Aiming to prove the advantage of our in situ synthesis, we applied in situ seeded AuNSt growth on gelatin hydrogels using PBS as the growth medium (instead of MilliQ water). In situ synthesis in PBS appeared to favor the growth of AuNSt with high extinction in the NIR after removing TX completely (Figures 7a and S25). The sample with the highest NIR extinction featured a tip‐to‐tip diameter of 84 ± 19 nm (corresponding to 0.2 µM HAuCl_4_ during seeding; Figures S26 and S27). Whereas colloidal surfactant‐free AuNSt are prone to reshaping and aggregation, in situ prepared surfactant‐free AuNSt are bound to the supporting hydrogel and show no signs of aggregation or significant reshaping even after incubation in PBS for 3 days (Figure S28). Of note, the peak corresponding to the core plasmon mode of the AuNSt is less prominent (Figure 7a,b) than that for the substrates grown with the standard growth solution (Figures 2 and 4, 5, 6). This observation likely relates to the slight differences in morphology of the nanostructures. Specifically, the AuNSt prepared in PBS have a larger core diameter (58 ± 11 nm) compared to those obtained with the standard growth solution containing TX (30 ± 8 nm), which would lead to a redshift. Furthermore, the spectral contribution by isotropic byproducts may also be lower for AuNSt prepared in PBS, owing to the higher shape yield (∼82% for the standard growth with TX and ∼90% for the surfactant‐free growth in PBS). Another contributing factor could be that the AuNSt grown in PBS has significantly higher polydispersity in branch dimensions, which would contribute to broadening of the plasmon mode in the NIR. However, variability in branch dimensions was comparable: 41 ± 13 nm (±32%) and 30 ± 8 nm (±27%) for the AuNSt grown in TX solution and in PBS without surfactant, respectively.

The shape of the surfactant‐free particles prepared in PBS was tuned similarly to nanostructures synthesized in water (Figures 7b–d and S29). Viability tests with MDA‐MB‐231 breast cancer cells showed that the biocompatibility of the nanostructures prepared in PBS was far superior to those prepared using aqueous TX solution. In situ growth was performed on Gel‐Alg hydrogels using either the standard preparation with TX in water or via surfactant‐free growth in PBS (yielding AuNSt with tip‐to‐tip dimensions of 93 ± 30 nm, Figures S30 and S31). Here, Gel‐Alg was used (instead of gelatin) because of its ability to maintain its structure in biological media and at 37 °C (Figure S31). As shown in Figure 7e, the viability of MDA‐MB‐321 cells was high when they were cultured in 2D (on a cell culture plate) and on plain Gel‐Alg. When the cells were cultured on Gel‐Alg with TX‐coated AuNSt, their viability decreased significantly. The viability was improved to ∼90% on TX‐coated AuNSt following a rigorous washing protocol with (4‐(2‐hydroxyethyl)‐1‐piperazineethanesulfonic acid) (HEPES) buffer (5 × washes of 1 h each). Alternatively, fluorescence microscopy gave excellent viability (>95%) for hydrogels carrying surfactant‐free AuNSt prepared with PBS as the growth medium (Figures 7e,f and S31).

## Conclusions

We have demonstrated the synthesis of gold nanoparticles directly on the surface of gelatin‐based hydrogel substrates via in situ seed‐mediated growth without using any colloidal components or self‐assembly steps. The seeds were prepared in two steps: (I) gold precursor binding to the gels then (II) reduction by incubation in NaBH_4_ solution. Both steps were required for overgrowth into AuNSt. In sum, the conditions of the seeding process could be used to modulate the morphology of the final nanostructures, from isotropic to anisotropic products as HAuCl_4_ concentration is decreased, in turn tuning the LSPR from ∼530 nm and into the NIR. While our implementation of the seed‐mediated growth process in situ represents an important step forward, in situ growth of various shapes with high yield and uniformity is still lagging behind colloidal synthesis. As an example, for AuNSt, it may be possible to precisely control the number of branches or their lengths by modifying the seeding conditions, i.e., via the implementation of different capping ligands, aging steps, etc., similar to what is attainable for colloidal AuNSt.^[^ ^9^ ^]^ Further study is also required to unlock the possibility to prepare other shapes by in situ seeded growth (e.g., nanorods, nanotriangles, cages, etc.) for improved sensing and to enable broader applications. Therefore, additional focus should be placed on developing these areas in future studies.

The interaction of Au^3+^ ions with various hydrogels, incorporating different polymer concentrations, ionic, and covalent crosslinking mechanisms, was studied by means of UV–vis spectroscopy, ICP‐MS, NMR, and FTIR spectroscopies. It was found that the presence of gold‐binding functional groups within the gels best promotes in situ growth. In addition, both secondary nucleation and other unwanted side reactions can inhibit in situ seeded growth. For secondary nucleation, HCl addition improves AuNSt yield. In ionically linked alginate‐based gels, proper curing is important to prevent the reduction of gold ions outside of the intended growth scheme, and extra washing steps hinder side reactions between the gold precursor and the curing agents in covalently photocrosslinked gels. The diverse compositions tested herein have variable physical characteristics and degradation profiles, which is important for applications such as 3D bioprinting and for maximizing substrate compatibility with different cell types. While the results on PEGDA hydrogels indicate the need for additional systematic studies to translate *in* *situ* growth to hydrogels without peptide bonds, carboxyl, hydroxyl, or amide groups, our results point toward future possibilities to apply in situ growth to peptides containing these moieties (e.g., collagen and Fmoc‐dipeptides).

Harnessing the ability to perform seed‐mediated in situ growth on different gel formulations, we demonstrated that in situ seeded growth enables straightforward preparation of biocompatible nanostructures under unconventional conditions. Specifically, surfactant‐free AuNSt can be grown using a high ionic strength solution (PBS) as the growth medium. Even without the use of surfactants, the shape of the particles can be tuned through the seeding conditions. Translating the surfactant‐free synthesis in PBS to Gel‐Alg allowed us to perform biocompatibility tests with MDA‐MB‐231 breast cancer cells, which is relevant for in vitro cell culture models. We found that AuNSt prepared in PBS without surfactant yields excellent biocompatibility, whereas those prepared using TX require thorough washing to attain high cell viability. This improvement in biocompatibility has relevant implications for surface‐enhanced Raman scattering (SERS)‐based (bio)sensing. Moreover, sensitivity may also be improved by avoiding the use of surfactants, which are known to (partly) block the nanoparticle's surface.^[^ ^67^, ^77^, ^78^, ^79^ ^]^ All in all, we gain a deeper understanding of the relevant chemical interactions affecting in situ growth on hydrogels, resulting in a more robust and translatable seeded in situ growth protocol for the fabrication of plasmonic‐hydrogel hybrid biomaterials with potential applications as labeled or label‐free biosensors to track small molecules, therapeutics, or biomarkers.

## Supporting Information

Full details on the materials, experimental methods, and characterization used. Additional UV–vis and electron microscopy characterization of the in situ grown nanostructures, rheological characterization of the hydrogels, full data on the gold ion‐hydrogel UV–vis binding experiment, characterization for the AuNSt synthesis on PEGDA, additional data on the optimization of AuNSt growth on hydrogels with different polymer contents and compositions, and additional characterization for the AuNSt growth in PBS and the corresponding cell viability studies.

## Conflict of Interests

The authors declare no conflict of interest.

## Supporting information



Supporting Information

## Data Availability

The data that support the findings of this study are available from the corresponding author upon reasonable request.
