# Supplementary material for: Plasmonic‐Hydrogel Hybrid Biomaterials Via In Situ Seeded Growth
Source: Angew Chem Int Ed Engl. 2025 Apr 22;64(25):e202501854. doi: 10.1002/anie.202501854 (PMC12171314; doi:10.1002/anie.202501854)
Supplement: Supplementary file 1 — Supporting Information [file ANIE-64-e202501854-s001.docx]

**Supporting Information for:**

**Plasmonic-Hydrogel Hybrid Biomaterials *via In Situ* Seeded Growth**

Gail A. Vinnacombe-Willson,^a,b*^ Manuel Núñez-Martínez,^a^ Ada Herrero-Ruiz,^a,b^ Francisco Bevilacqua,^a^ Raquel Pazos,^a^ Lara Troncoso-Afonso,^a,b,c^ Marta Gallego-González,^a^ Leonardo Scarabelli,^d^* and Luis M. Liz-Marzán^a,b,e,f^*

1. CIC biomaGUNE, Basque Research and Technology Alliance (BRTA), 20014 Donostia-San Sebastián, Spain
2. Centro de Investigación Biomédica en Red, Bioingeniería, Biomateriales y Nanomedicina (CIBER-BBN), 20014. Donostia-San Sebastián, Spain
3. Department of Applied Chemistry, University of the Basque Country, 20018 Donostia-San Sebastián, Gipuzkoa, Spain
4. Department of Chemistry and Process & Resource Engineering, ETSIIT, University of Cantabria, Av. de los Castros, s/n, 39005 Santander, Spain
5. Ikerbasque, 43009 Bilbao, Spain
6. CINBIO, Universidade de Vigo, 36310 Vigo, Spain

*Corresponding authors’ email: [gvinnacombe@cicbiomagune.es](mailto:gvinnacombe@cicbiomagune.es) (G. A. V.-W.); [leonardo.scarabelli@unican.es](mailto:leonardo.scarabelli@unican.es) (L. S.); [llizmarzan@cicbiomagune.es](mailto:llizmarzan@cicbiomagune.es) (L. M. L.-M.)

**CONTENTS:**

**MATERIALS & METHODS**

**Materials**

1. **Hydrogels**
2. **Nanoparticle synthesis**
3. **Cell culture**

**Methods**

1. ***In Situ* Synthesis of AuNSt**

Seeding in water

Growth in aqueous medium

Seeding and growth in PBS

1. **Synthesis of GelMA**
2. **Hydrogel preparation**
3. **Characterization**

I. Fourier transform-infrared spectroscopy (FTIR)

II. Proton nuclear magnetic resonance (^1^H-NMR)/Correlated spectroscopy (COSY)

III. Scanning electron microscopy (SEM)

IV. Transmission electron microscopy (TEM) and high-angle annular dark-field scanning transmission electron microscopy (HAADF-STEM)

V. Optical characterization and UV-vis spectroscopy gold binding study

VI. Inductively coupled plasma-mass spectrometry (ICP-MS)

VII. Rheology and swelling

**ADDITIONAL FIGURES**

**A. Additional characterization for Au nanoparticles grown in situ on gelatin 10% w/v**

**B. Additional spectroscopic characterization of gold ion interactions with hydrogels**

**C. Rheological and swelling properties of hydrogels**

**D. UV-vis spectroscopy gold binding experiment**

**E. AuNSt synthesis on PEGDA**

**F. Additional data for the optimization of AuNSt growth on hydrogels with different polymer content and composition**

**G. Additional characterization for the growth of AuNSt in PBS and cell viability studies**

**REFERENCES**

**MATERIALS & METHODS**

**Materials**

1. **Hydrogels**

Gelatin from porcine skin (gel strength 300, Type A; CAS: 9000-70-8), 2-hydroxy-4′-(2-hydroxyethoxy)2-methylpropiophenone (irgacure; Ig, 98%; CAS: 106797-53-9), methacrylic anhydride (with 2,000 ppm topanol A as inhibitor, ≥94%; CAS: 760-93-0), lithium phenyl-2,4,6-trimethylbenzoylphosphinate (LAP, ∙≥ 95%: CAS: 85073-19-4), poly(ethyleneglycol) diacrylate (PEGDA, average Mn 700; CAS: 26570-48-9), deuterium oxide (D_2_O, ≥99%; CAS: 7789-20-0), calcium chloride (CaCl_2_, ≥96.0%; CAS: 1003-52-4)and alginic acid sodium salt (sodium alginate, CAS: 9005-38-3) were all purchased from Merck.

1. **Nanoparticle synthesis**

Analytical grade reagents, HAuCl_4_⋅3H_2_O (≥99.9%, CAS: 16961-25-4), silver nitrate (AgNO_3_; ≥99.9%, CAS: 7761-88-8), l-ascorbic acid (99%, CAS: 50-81-7), hydrochloric acid (HCl; ACS reagent 37%, CAS: 7647-01-0), Triton X-100 (TX, laboratory grade; CAS: 9002-93-1), 4-mercaptobenzoic acid (99%, CAS: 1074-36-8), sodium borohydride (NaBH_4_, 99%, CAS: 16940-66-2), cetyltrimethyl ammonium chloride (CTAC, 25% wt, CAS: 112-02-7), and 200 proof ethanol (≥99.8%, CAS: 64-17-5), were purchased from Merck.

Phosphate buffer saline (PBS) was prepared from sodium chloride (NaCl, ≥99.5%; CAS: 7647-14-5), potassium chloride (KCl, ≥99%; CAS: 7447-40-7), sodium diphosphate (Na_2_HPO_4_, ≥99%; CAS: 7558-79-4) and potassium phosphate monobasic (KH_2_PO_4_, ≥99%; CAS: 7758-11-4), all of which were purchased form Merck.

All glassware, lids, and magnetic stir bars used for synthesis were cleaned thoroughly with *aqua regia* (3:1 concentrated HCl to HNO_3_ (68%, CAS: 7697-37-2)) and rinsed thoroughly with MilliQ water three times before use. Warning: *aqua regia* is extremely corrosive and causes severe burns. Care is required during its preparation and usage in a fume hood.

1. **Cell culture**

MDA-MB-231 cells were purchased from American Type Culture Collection (ATCC, HTB-26). Dulbecco's modified eagle medium (DMEM), fetal bovine serum (FBS), and penicillin-streptomycin (PS) were purchased from Invitrogen. FBS and PS were used at 10% and 1% respectively to prepare complete DMEM (cDMEM). All cell line cultures were grown in standard tissue culture conditions at 37 °C with 5% CO_2_. Collagen type I (rat tail high concentration; REF: 354249) and fibronectin (human, natural; REF: 354008) were purchased from Corning.

**Methods**

1. ***In Situ* Synthesis of AuNSt**

**Seeding in water:** The seed-mediated growth of AuNSt *in situ* was carried out following the schematic in **Figure 1A** in the **Main Text**. All steps were carried out at room temperature unless otherwise stated. Therefore, during all synthesis steps, the gels were in their gelated states (not liquid). First, the gels (prepared in 200 µL aliquots) were incubated under mild stirring (500 rpm) for 5 min in 5 mL of MilliQ water with 0.2 – 3.0 µM (2 – 30 µL of 0.5 mM) HAuCl_4_ or 0.1 – 0.9 mM (10 – 90 µL of 50 mM) HAuCl_4_, as indicated. The standard growth condition comprised 0.2 µM (2 µL of 0.5 mM) HAuCl_4_/5 mL. The samples were rinsed three-fold with 3 – 5 mL of MilliQ water, then the sample was placed in 4.7 mL MilliQ water under rapid stirring (1000 rpm), to ensure uniform nucleation of the seed particles, similar to the established processes for colloidal synthesis, then 300 µL of freshly prepared 10 mM NaBH_4_ was added rapidly with one fast injection to the solution (0.6 mM final concentration). The hydrogels were left stirring at 1000 rpm for 10 min. After this step, small gold nanoparticle seeds should be formed. Then the solution was removed from the vial and replaced with fresh MilliQ water, and the samples were stirred at 250 rpm for another 20 min before continuing to the growth step, to ensure the removal of excess NaBH_4_. Finally, the samples were rinsed 2 more times prior to overgrowth.

**Growth in aqueous medium:** The AuNSt growth solution was prepared similar to previous reports on colloidal synthesis.^1,2^ 50 µL HAuCl_4_ 50 mM (0.5 mM final concentration), 50 µL AgNO_3_ 10 mM (0.1 mM final concentration), and 125 – 150 µL HCl 1M (25 – 30 mM final concentration; “standard” conditions applied 30 mM HCl) were added to 5 mL of 100 mM aqueous TX under mild stirring (250 rpm). Then, the stirring speed was increased to 1000 rpm, and quickly, 160 µL AA 100 mM (3.2 mM final concentration) was added under one fast injection. After 10 – 15 s, the solution changes from yellow to clear as Au^III^ is reduced to Au^I^. The hydrogel substrate containing gold seeds was added immediately to the growth solution once the color of the solution turned colorless. The stirring was maintained at 1000 rpm for 5 min. Depending on the conditions of the seeding step and which hydrogel formulation is used, the color of the final hydrogels ranges from bright pink through purple to dark blue-black (from more isotropic to more anisotropic products, respectively, see **Figures 2** and **7** in the **Main Text**). The growth solution was removed from the vial and the hydrogel was washed 3 times with MilliQ water.

Gold nanospheres were grown following the same exact process as for AuNSt, just replacing the growth solution with one containing 5 mL of 50 mM cetyltrimethylammonium chloride (CTAC) as the surfactant, 0.5 mM HAuCl_4_ (50 µL of 50 mM solution), 2 mM HCl (10 µL of 1 M solution), and 0.6 mM ascorbic acid (30 µL of 100 mM solution; added last, immediately prior to the addition of the seed-containing gel). The plasmonic response of all grown samples was finally analyzed with UV-vis spectroscopy and their morphology evaluated with scanning electron microscopy.

**Seeding and growth in PBS:** A 500 mL of a 1× PBS solution was prepared containing 20 g NaCl, 0.5 g KCl, 2.8 g Na_2_HPO_4_, and 0.51g KH_2_PO_4_. The seeds were prepared in the same way as in aqueous medium, adding 0.2 – 3.0 µM (2 – 30 µL of 0.5 mM) HAuCl_4_ to 1× PBS under mild stirring (500 rpm) for 5 min, rinsing the sample 3 times with 1× PBS, then replacing the gel in 4.7 mL 1× PBS and rapidly adding 300 µL 10 mM freshly prepared NaBH_4_ under rapid stirring (1000 rpm) for 10 min (0.6 mM final concentration). The solution was replaced with 1× PBS and the samples were aged for another 20 min prior to growth. Before the growth step, the samples were rinsed in 1× PBS two more times. The growth in PBS was performed in the same way as the growth in aqueous medium, but using 50 µL HAuCl_4_ 50 mM (0.5 mM final concentration), 50 µL AgNO_3_ 10 mM (0.1 mM final concentration), 150 µL HCl 1M (30 mM final concentration) in 5 mL of 1× PBS solution with 0-100 mM TX, as indicated in the text (standard conditions comprised 5 mL of 1× PBS with 0 mM TX), and 160 µL AA 100 mM (3.2 mM final concentration). The hydrogels were characterized with UV-vis spectroscopy and electron microscopy.

1. **Synthesis of GelMA**

Gelatin methacryloyl (GelMA) was prepared by dissolving 5 g of gelatin type A from porcine skin in 100 mL of 1× PBS solution at 50 °C, adapted from previous works.^3^ After gelatin is fully dissolved (~20 min), 4 mL of maleic anhydride was added quickly under stirring (1000 rpm). The solution was left to stir at 50 °C for 4 hours. The solution was then purified by dialysis against water for 5 d (45 °C, 400 rpm stirring), the water being replaced twice per day (usually at 6-8 h intervals). After 5 d, the solution was collected and frozen overnight at -18 °C, then isolated by freeze drying over ~3 d. The final solid was characterized by ^1^H-NMR (see characterization section).

*N.B*.: Methacrylic acid ((^1^H-NMR (500 MHz, D_2_O): δ 1.97 (s, 6H), 6.00 (d, 2H, J = 6.18 Hz), 6.35 (d, 2H)) produced as a by-product of the reaction or excess of methacrylic anhydride should not be present in high amounts to avoid toxicity problems. If there is a significant quantity of methacrylic acid, longer dialysis times or more frequent water replacement during dialysis may be required.

1. **Hydrogel preparation**

Gelatin and Gel-Alg solutions were prepared by dissolving 100 mg/mL gelatin or 100 mg /mL gelatin and 20 mg/mL sodium alginate, respectively, in MilliQ water or PBS. PEGDA solutions were prepared by dissolving 100 µL/mL in MilliQ water along with 0.5% *w/w* photoinitiator Ig or 0.15% *w/w* photoinitiator LAP. GelMA solutions were prepared by adding 10% *w/w* freeze dried GelMA to MilliQ water along with 0.5% w/w photoinitiator irgacure or 0.15% photoinitiator LAP. All solids were allowed to completely dissolve by placing the solutions in an oven at 70 °C for ~30 min, and all samples were protected from light using aluminum foil during dissolution. Once fully dissolved, ~200 µL of each solution was added into a square or circular well with lateral side or diameter of ~1 cm (*i.e.*, in a 48-well plate). Physical hydrogels of gelatin and Gel-Alg were prepared by cooling the 200 µL aliquots at 4 °C for 10 min, then bringing them back to room temperature, prior to AuNSt growth. Gel-Alg underwent an additional curing step forming ionic bonds between hydroxyl and carbonyl groups of alginate and M^2+^ ions.^4^ Namely, Gel-Alg gels were incubated in fresh 100 mM CaCl_2_ solution immediately before AuNSt synthesis. The aliquots of GelMA and PEGDA were subsequently cured with a UV lamp (Vilber Lourmat, VL-230, 30 W, 365 nm) for 30 min and 2 min, respectively.

*N.B*.: Depending on the UV lamp and wavelength, the curing process might differ. Should challenges with proper curing arise, make sure that the container has a configuration that allows for light penetration to all areas of the pre-polymer.

1. **Characterization**

**I. Fourier transform-infrared spectroscopy (FTIR)**

FTIR was used to interrogate the specific chemical interactions between hydrogels and gold ions. The as-prepared gels were incubated in separate 1 mL aliquots of 50 mM aqueous HAuCl_4_ for 5 min, then rinsed thoroughly with MilliQ water three-fold. The gels were immediately flash frozen in liquid nitrogen and lyophilized for 24 h. The samples were protected from light using aluminum foil as much as possible during the entire process, to prevent the reduction of Au^3+^ by amides. Finally, an attenuated total reflectance (ATR)-FTIR Bruker INVENIO X spectrometer equipped with an accessory featuring a single-reflection monolithic diamond for measuring ATR was used to obtain the spectra of the gels. A small amount of the freeze-dried sample was placed on the ATR crystal, fully covering its surface. Background correction was performed by the software automatically and spectra were collected from 4000 to 400 cm^-1^ with 31 scan accumulation.

**II. Proton nuclear magnetic resonance (^1^H-NMR)/Correlated spectroscopy (COSY)**

The different hydrogels as well as their chemical interactions with gold ions were studied using ^1^H-NMR and COSY spectroscopy using a Brucker AVANCE III 500 MHz. 1 mg/mL of each polymer was dissolved in D_2_O (residual peak at 4.79 ppm), 64 scan accumulation.

The ^1^H-NMR spectra were acquired with water signal suppression, with the residual signal of deuterated water (D₂O) referenced to 4.79 ppm. Chemical shifts are reported in ppm (δ) and splitting patterns are designated as “d”, doublet, “dd” double doublet and “m”, multiplet. For complete characterization, two-dimensional experiments, *i.e.*, COSY, were also used. All spectra were acquired in D_2_O (1 mg/mL) at 37 °C to prevent physical gelation, except for PEGDA, which was recorded at room temperature (it is liquid at room temperature). To evaluate the hydrogel-gold interactions, spectra of the polymer solutions were obtained upon addition of 4 µL/mL 50 mM aqueous HAuCl_4_ (at 37 °C, with the exception of PEGDA). All spectra were analyzed using MestRenova software.

For GelMA, ^1^H-NMR spectra were also used to estimate the methacrylation degree (**Table S1**, **Figure S19**). The resulting spectra showed the characteristic peaks of the gelatin backbone as well as for the vinyl moiety ($\delta$= 5.45 and 5.69 ppm) and the methyl group ($\delta$=1.95 ppm) belonging to the methacrylate group. The degree of methacrylation (~80% batch-to-batch) was determined by comparing the signal of phenylalanine protons with the signal of the vinylic protons of the methacrylate group. Considering the proportion of phenylalanine residues and lysine residues, the degree of methacrylation can be calculated according to **Eq. S1**:

$Degree of methacrylation \left( \% \right)=\frac{\int Vinylic H}{\int Phe H} \times100=\frac{\int Vinylic H}{5} \times100$ **Eq. S1**

| Table S1. Integrated peak area for key ^1^H-NMR peaks. | | |
| --- | --- | --- |
| Batch | $\int\boldsymbol{Vinylic H}$ | **Degree of methacrylation (%)** |
| 1 | **3.9** | **78** |
| 2 | **4.22** | **84.4** |
| 3 | **3.39** | **78.6** |
| Average |  | **80 ± 4** |

**III. Scanning electron microscopy (SEM)**

SEM images of vacuum-dried hydrogels (using a desiccator for 48 h) were obtained with a JSM-IT800HL from JEOL (Tokyo, Japan), equipped with a backscatter electron detector (BSE detector, scintillator-photomultiplier detector design) using 3-5 kV, under high vacuum.

**IV. Transmission electron microscopy (TEM) and high-angle annular dark-field scanning transmission electron microscopy (HAADF-STEM)**

TEM images were collected with a JEOL JEM-1400PLUS (Tokyo, Japan) microscope operating at 80 kV. The AuNSt grown on gelatin were collected by dissolving the gel at 37 °C for 30 min (or until fully dissolved), then washing the particles by centrifugation. The round of centrifugation was performed at 37 °C and 8000 rpm (7.08 × 10^4^ g) for 15 min. The particles were redispersed in 1 mL MilliQ water using sonication, then washed 2 more times at 8000 rpm (7.08 × 10^4^ g) for 15 min. After the final wash, the particles were resuspended in 100 µL of MilliQ water, and 3-5 µL of the solution was drop casted on a TEM grid.

For the seeds, an additional silver overgrowth step was performed to increase their overall size and protect them during the heat treatment/dissolution of gelatin at 37 °C. The hydrogels with seeds were immersed in 5 mL of MilliQ water, and a syringe pump was used to add 10 mM aqueous AgNO_3_ and 10 mM aqueous AA to the solution at a rate of 50 µL/min. The addition of reagents was performed over the course of 1 h. During the addition of AgNO_3_ and AA, the solution containing the gel was replaced with MilliQ water after 30 min to help prevent secondary nucleation of silver particles on the hydrogel/in the solution. Ag-coated seeds were collected in a similar manner as for *in situ* grown AuNSt: gelatin was dissolved at 37 °C for 30 min, then centrifuged at 37 °C at 18000 rpm (3.59 × 10^5^ g) for 60 min. Then, they were redispersed in 1 mL of MilliQ water and centrifuged at 18000 rpm (3.59 × 10^5^ g) for 60 min twice more. After the final centrifugation, the seeds were redispersed in 50 µL of MilliQ water. The grids were prepared by drop casting 3-5 µL of the obtained solution (silver-coated seed characterization in **Figure S2**).

*N.B.:* Excess organic contaminants can be removed by rinsing the grid gently with 100 µL of MilliQ water at 37 °C. This was done prior to taking HAADF-STEM measurements.

HAADF-STEM images were acquired on a JEOL JEM-2100F UHR (Tokyo, Japan) microscope at 200 kV in scanning mode, with a probe size of 0.7 nm and a camera length that ensures an inner detector angle of 60 to 160 mrad (HAADF) to observe the core-shell nature of silver-coated gold seeds (confirming the formation of seeds, **Figure S2**).

**V. Optical characterization and UV-vis spectroscopy gold binding study**

UV-visible spectra of the gels and collected nanoparticles were recorded in an Agilent 8453 UV-visible-NIR photodiode array spectrophotometer using plastic cuvettes (*l*=1 cm). The gold binding experiment was performed in a quartz cuvette (5 mm, Art. No. 100-5-40; Hellma Analytics) with a UV-visible-NIR CARY 5000 Varian spectrophotometer (to measure the spectra at 190 – 450 nm). The as-prepared hydrogels were incubated in a 5 mL aqueous solution containing 20 µL 50 mM HAuCl_4_. The hydrogels were removed from the solutions at different time points (2, 5, 10, 15, 20, 30, 40, 50, and 60 min) and the spectra of all solutions were measured to evaluate the amount of gold ions remaining in the solution (*i.e*., quantifying the ions that were *not* bound to the gels). Higher gold binding/greater affinity of the gels for gold ions was indicated by a greater increase in the absorbance of the collected incubation solution.

**VI. Inductively coupled plasma-mass spectrometry (ICP-MS)**

The hydrogels were analyzed by ICP-MS following 5 min incubation in a 50 mM HAuCl_4_ aqueous solution to compare the amount of gold ions sequestered by each formulation. Following incubation, the samples were rinsed well 3 times in MilliQ water, dried over 2 d under vacuum, then dissolved in 1 mL of *aqua regia* (3:1 concentrated HCl:HNO_3_) over 2 d. The samples were analyzed with an iCAP-Q ICP-MS (Thermo Scientific, Bremen, Germany) equipped with an autosampler ASX-500 (CETAC Technologies, Omaha, USA). The digested samples were then diluted in 1:100 diluted *aqua regia* (2% HNO_3_ / 0.5% HCl) prior to the ICP-MS measurements. Quantification was made through a calibration curve using a CMS2 reference solution (Inorganic Ventures, Lakewood, USA) for Au quantification. The dilutions of the reference solution were carried out in diluted *aqua regia* (2% HNO_3_ / 0.5% HCl) to obtain a medium with a similar composition as the digested samples. To improve linearity and more accurately determine detection and quantification limits, as well as to minimize possible instrumental errors, an internal standard (Indium 50 µg/L) was added to the calibration solutions, samples, and blanks. After analysis of the calibration solutions by ICP-MS, comparison between the intensity obtained from each sample and their theoretical concentrations has shown a linear range between 1 and 200 µg/L (R^2^>0.998). The ICP/MS acquisition parameters were optimized, and a 15 min equilibration and prior tuning were performed. Sample and calibration dilutions were prepared immediately prior to analysis. In addition, all the samples were measured in triplicate, obtaining a mean value ± standard deviation. The quantification of the Au was carried out using the Qtegra v2.6 software (ThemoFisher, Bremen, Germany) monitoring the isotope ^197^Au and ^115^In is an internal standard.

**VII. Rheology and swelling**

Rheological characterization was performed with a Physica MCR 302 rheometer (Anton Paar, Spain) using a sand blasted parallel plate (geometry PP08/P2, Ø = 8 mm) to prevent the substrates from slipping. The hydrogel disc was placed between the fixed plate and the measuring plate with a 2 mm gap.

Swelling tests in MilliQ water and in AuNSt growth solution were performed on the as-prepared gels immediately after curing. The weight of the hydrogels was measured at different time points after wicking away excess moisture using a paper towel. Swelling in water was evaluated at 5 min, 10 min, 30 min, 1 h, 2 h, 3 h, 1 d, and 6 d. Swelling during AuNSt growth solution was measured only at 5 min, which is the same as the duration of the overgrowth step. Beyond 5 min, nanoparticles also start to nucleate on the gels even in the absence of seeds, which might skew or otherwise affect the evaluation of the swelling due to screening effects. Thus, time points beyond 5 min were not considered.

**ADDITIONAL FIGURES**

**A. Additional characterization for Au nanoparticles grown in situ on gelatin 10% w/v**

Following the scheme in **Figure 1** of the **Main Text**, seeding was performed by first incubating the gel in gold precursor (**Step I**), then rinsing the sample and placing it in a solution with the strong reductant sodium borohydride (**Step II**) to achieve the complete reduction of Au^III^ to Au^0^, forming small nanoparticle “seeds.” Finally, after additional washing and sample aging steps (see **Methods Section A**), the samples are placed in a growth solution to obtain the final AuNSt (**Step III**). A number of control experiments to support the formation of seeds and their successful overgrowth were carried out (**Figure S1A**). Namely, performing: (i) only **Step I** and **Step III** (without **Step II**; “skip NaBH_4_”), (ii) only **Step II** and **Step III** (without **Step I**; “skip Au”), and (iii) only **Step III** (without **Step I** and **II**; “Growth only”). The spectra of the “all steps” sample confirm the presence of AuNSt, whereas the controls show very little growth overall (no spectroscopic indication of significant AuNSt growth) and minimal extinction at 400 nm (indicating a low presence of Au^0^).^5^ The “skip Au” sample had the highest extinction of all the controls, which could be due to effects from any residual active NaBH_4_ present in the gel. Longer aging times after **Step II** may reduce this unwanted nucleation, but aging periods longer than 30 min were avoided due to the known instability of seeds.^5–7^ Further supporting the formation of seeds, after completing **Step I** and **Step II**, silver coating was performed, resulting in yellow gels (**Figure S1B**). TEM and HAADF-STEM of the collected silver-coated seeds were used to estimate their dimensions (**Figure S1C, Figure S2**).

| 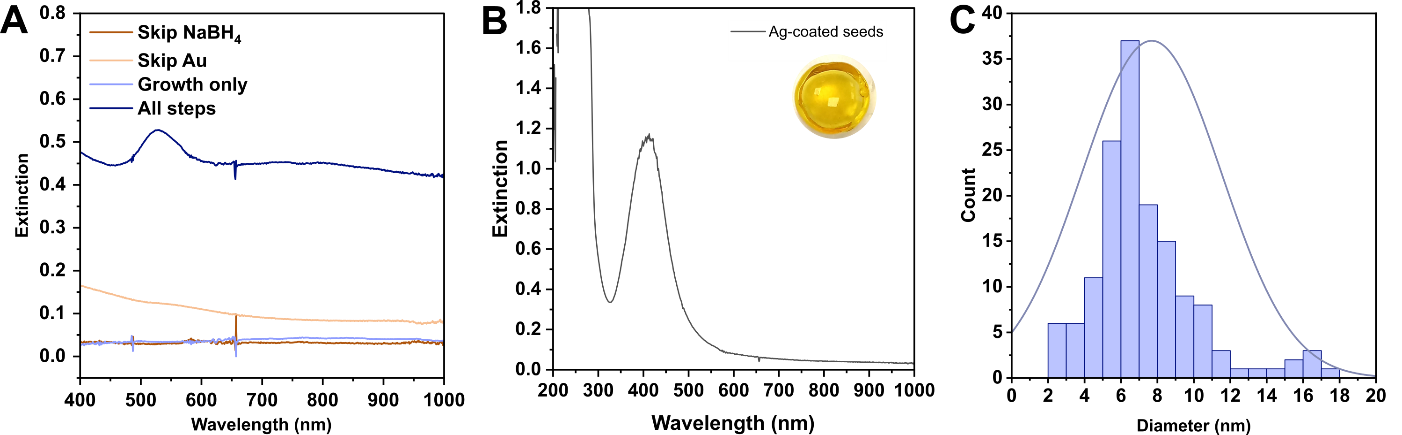 | |
| --- | --- |
| **Figure S1. A:** Control experiments showing that AuNSt are obtained only when all seeding steps (incubation with gold precursor, then incubation in NaBH_4_ solution) are performed. **B:** UV-vis spectra and digital photograph (inset) of silver-coated seeds. **C:** Size distribution of Au seed cores in the silver-coated seeds analyzed by HAADF-STEM; Ø = 8 ± 4 nm; n = 150 (**Figure S2**). | |
| 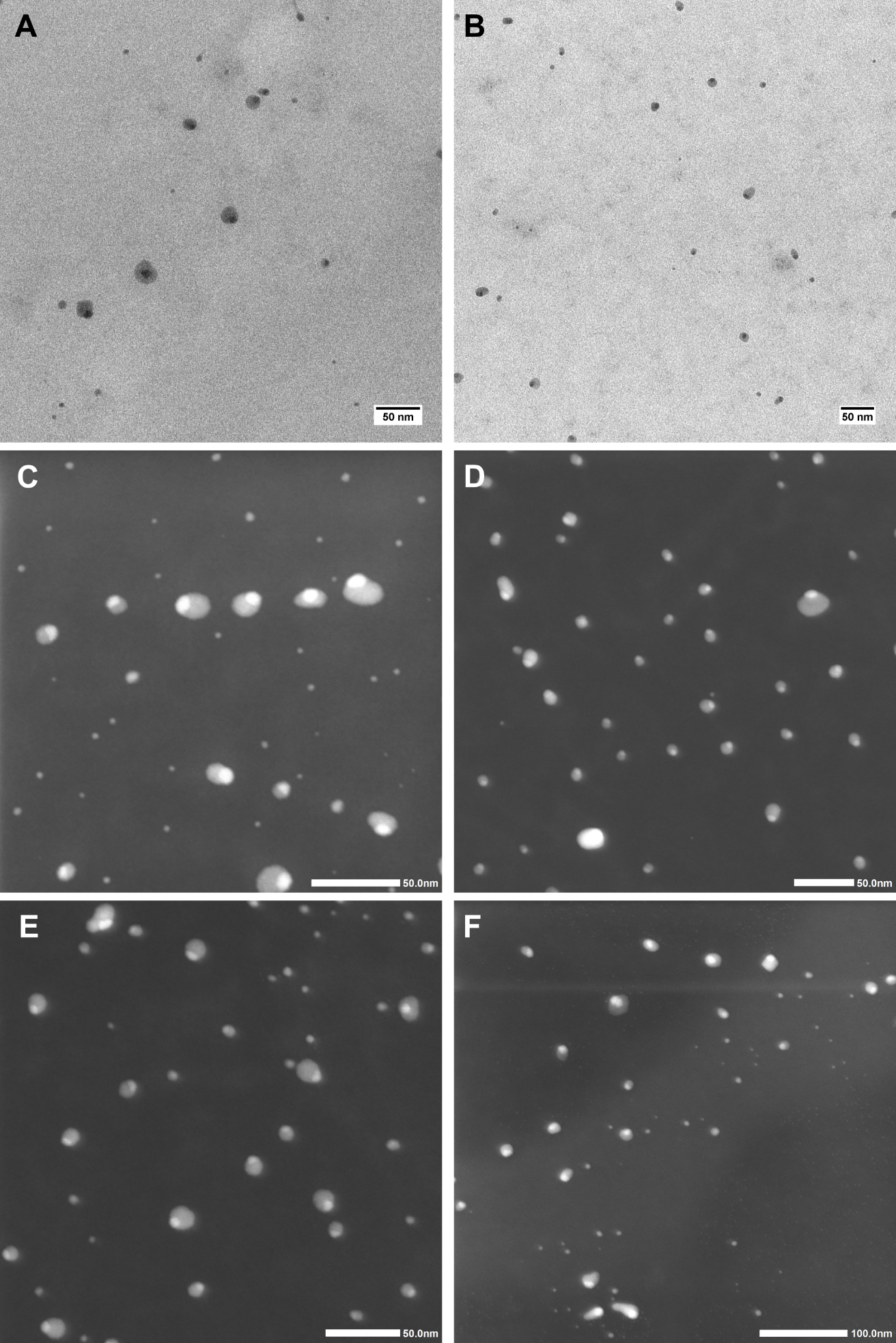 |  |
| **Figure S2. A,B:** TEM images of silver-coated seeds collected from the hydrogel (after dissolving gelatin). **C-F:** Corresponding HAADF-STEM images. |  |

Electron microscopy characterization was used to evaluate the morphology of the in situ prepared AuNSt. Gelatin can be dissolved at 37 °C, enabling collection of the nanoparticles and their analysis by TEM (**Figure S3A,B**).^8^ The AuNSt could also be observed with SEM (**Figure S3C**).

| 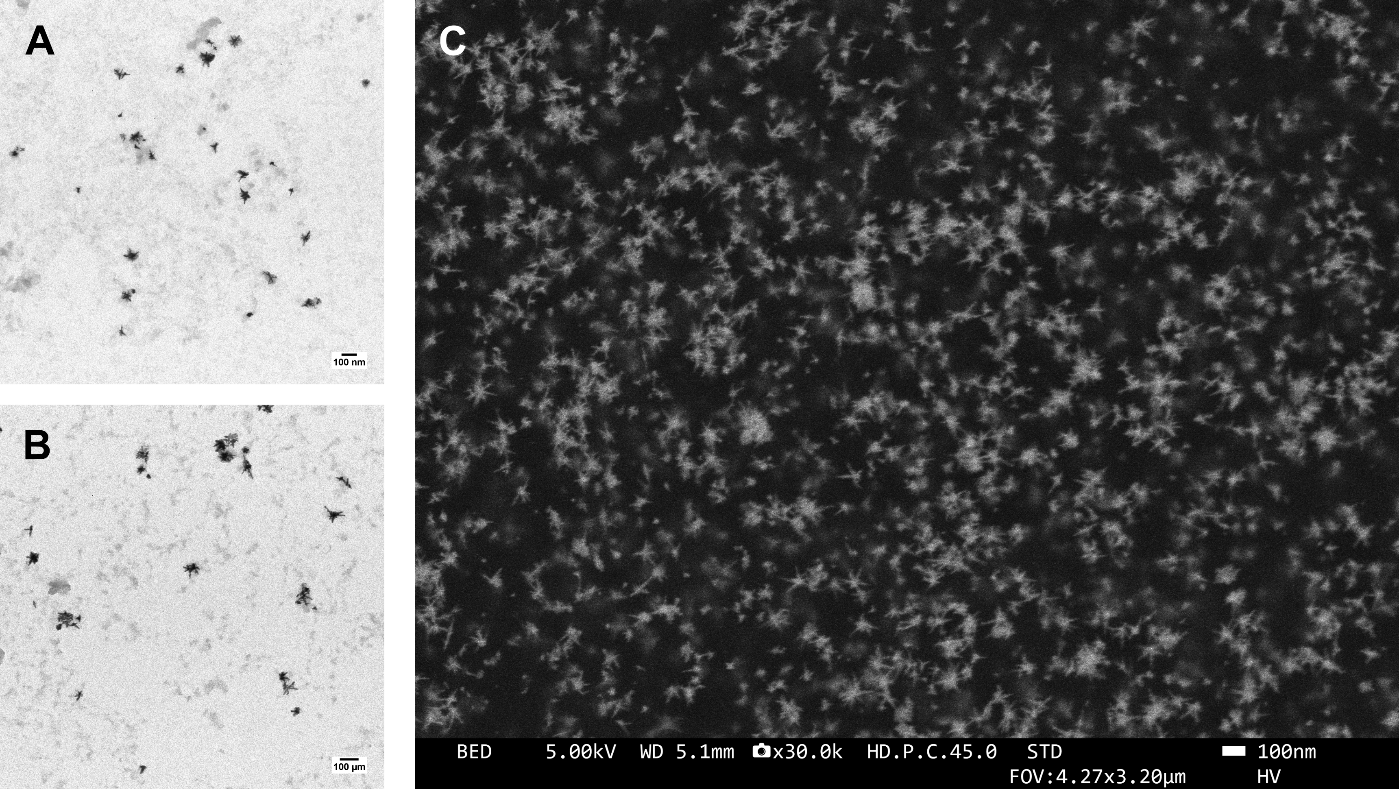 |
| --- |
| **Figure S3. A,B:** Additional TEM images of AuNSt collected after dissolving gelatin (10% w/v). **C:** Additional SEM image of the AuNSt on the dried gel (with vacuum desiccator). |

| 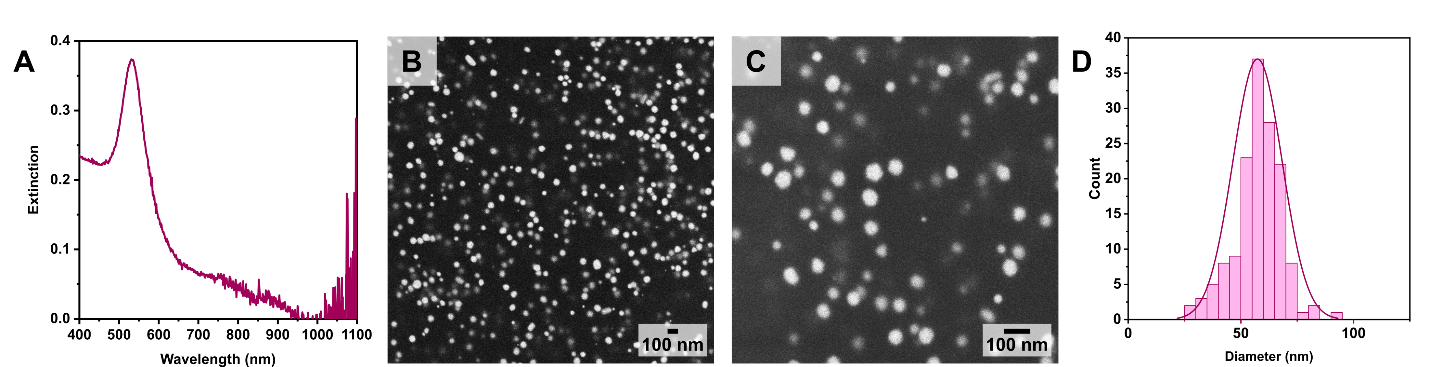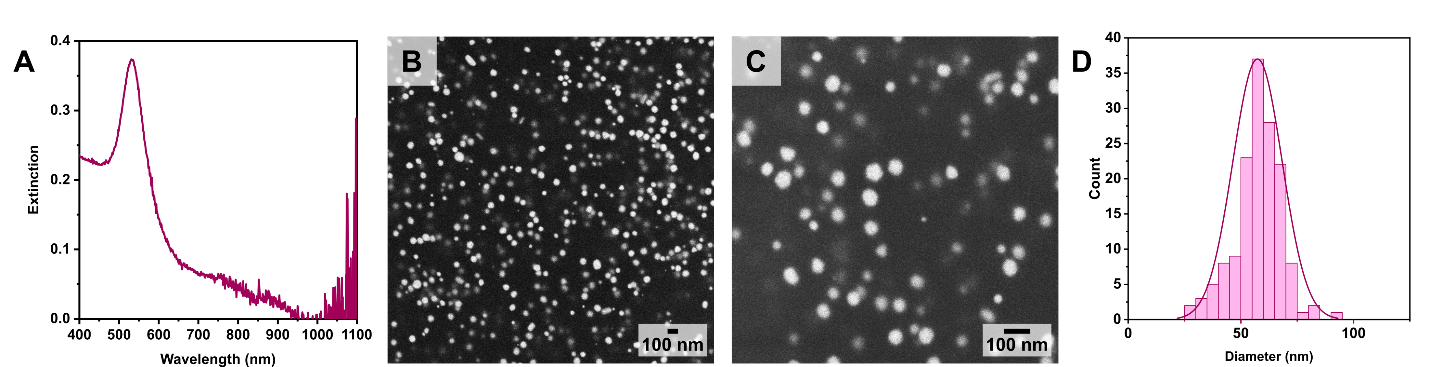 |
| --- |
|  |
| **Figure S4. A:** UV-vis spectrum of the obtained gold nanospheres (Ø = 57 ± 11 nm; aspect ratio: 1.1 ± 0.1; n = 150). **B,C:** SEM images of the gold nanosphere products. **D:** Size distribution of the products (n = 150). |

We sequestered the substrate from the growth solution at different time points to assess the effect of incubation time on the shape of the final AuNSt products (**Figure S5**).


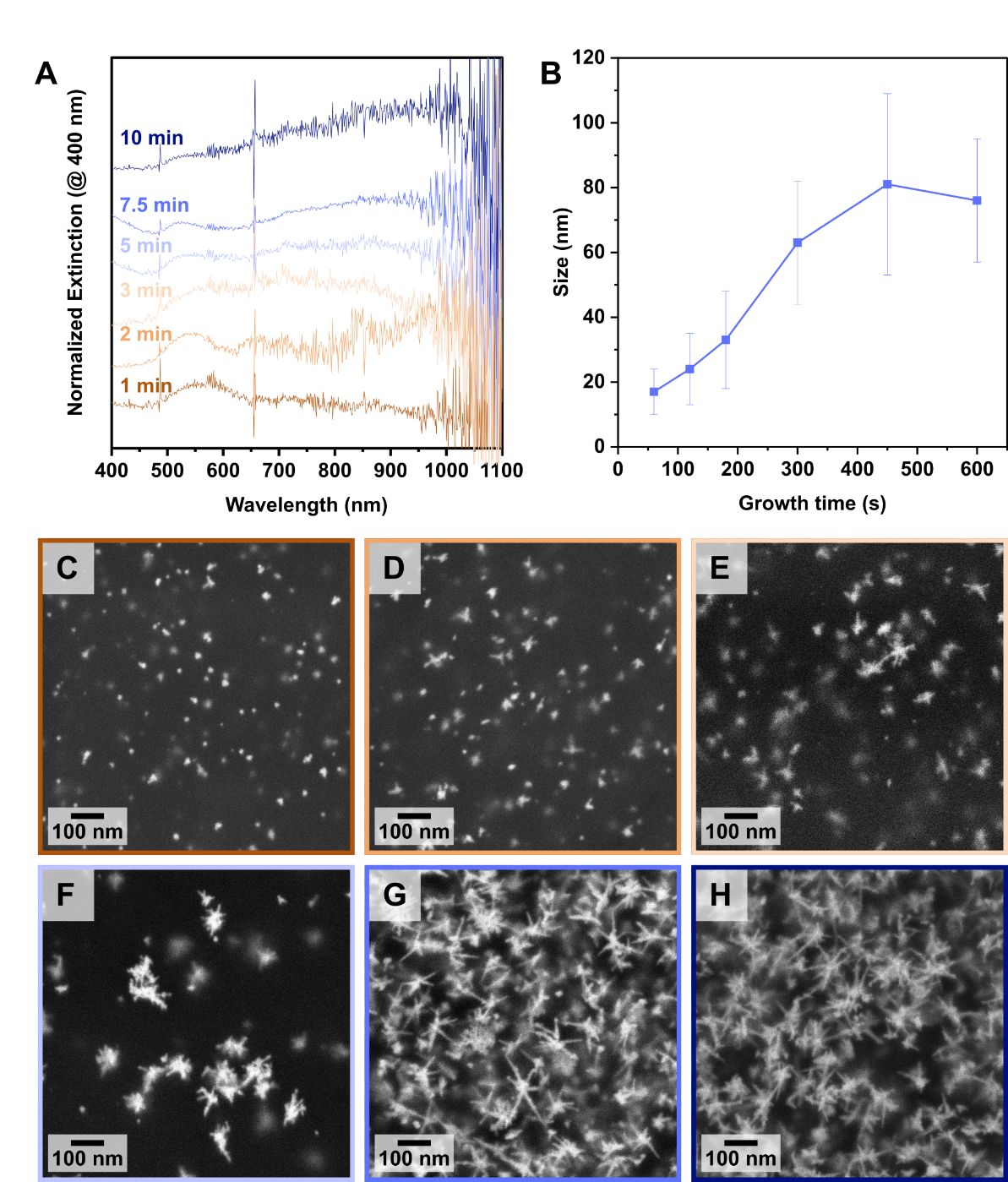


**Figure S5. A:** UV-vis spectra of the products obtained at different incubation times for Step III (the growth step; from **Figure 1A** in the Main Text). **B:** Trend in the size of the products at different time points, determined from the SEM images shown in panels **C-H**, corresponding to the selected time points.

From the data in **Figure S5**, we see that AuNSt continue to grow and increase in size during the first ~ 7 min of incubation, at which point the growth slows down. This could be, in part, because after ~8-10 min, secondary nuclei start to form and colloidal particles grow away from the surface in the growth solution; and as a result, they consume reagents that would contribute to the continued growth in situ.

In addition to growth time, we also interrogated the effect of stirring speed during the growth (**Figure S6**).


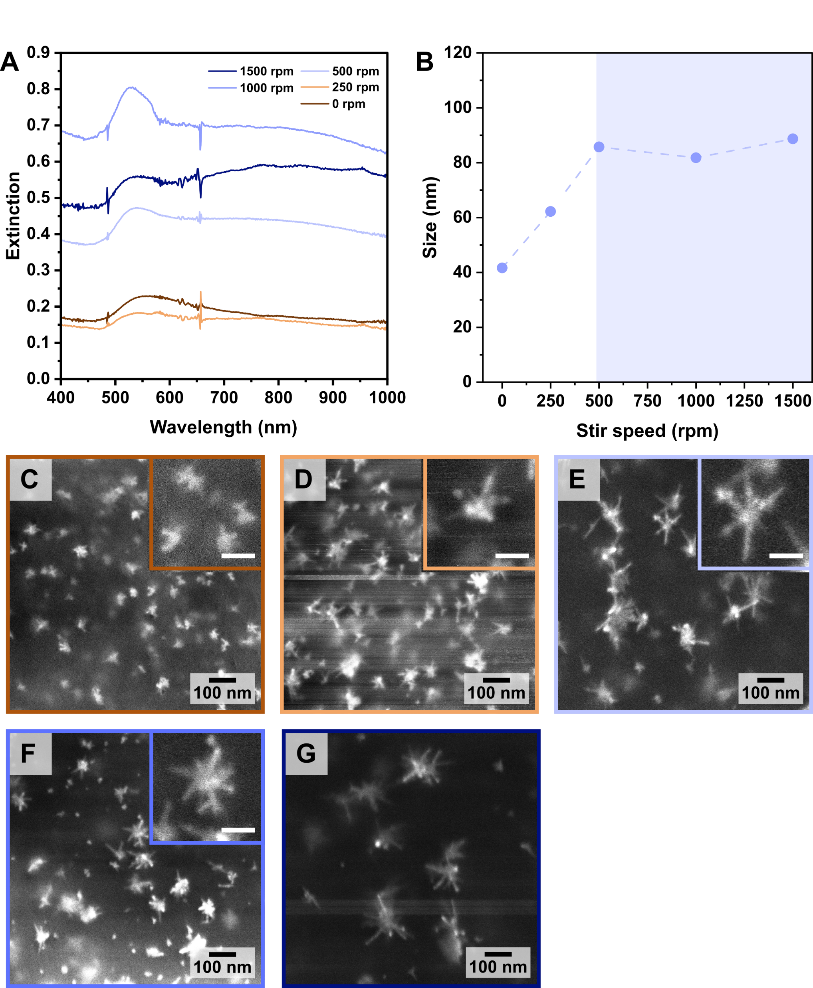


**Figure S6. A:** UV-vis spectra of the products obtained on 5% w/v gelatin after growth of AuNSt at different stirring speeds. **B:** Trend of the sizes of the products and **C-G:** SEM images showing their corresponding morphologies.

In colloidal seed-mediated growth, the seeding step is important for the overgrowth of anisotropic shapes, and the size and quality of the seeds (uniformity, crystal structure, *etc.*) influence the final products.^5–7^ We show here that varying the amount of gold precursor during the first seeding step, different size, shape, and density of the particles could be obtained, as shown by SEM (**Figure S7-S9**).

| 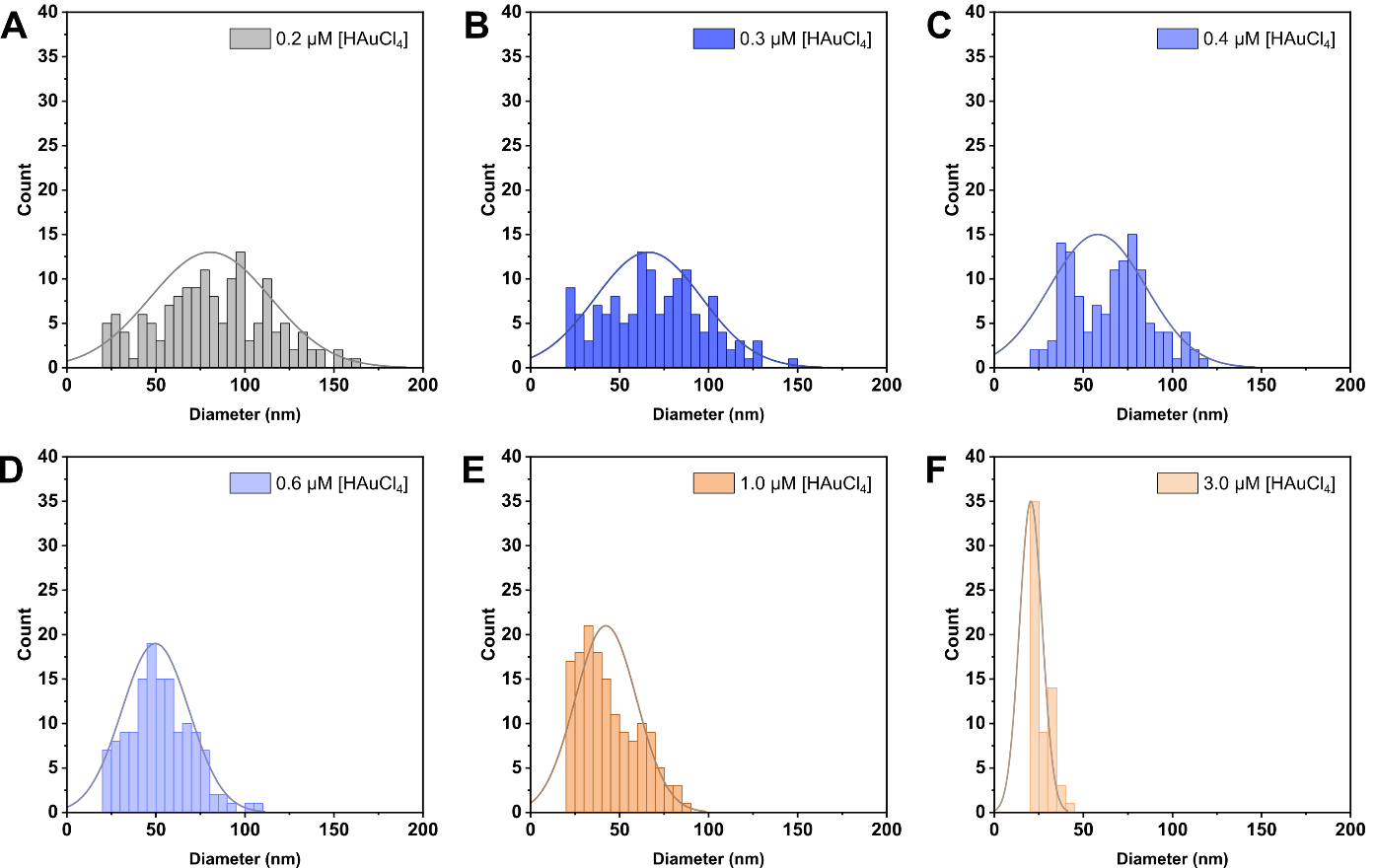 |
| --- |
| **Figure S7. A-F:** Size-distributions of the final overgrown nanoparticles obtained after changing the gold precursor concentration during **Step I** of the seeding (on 10% *w/v* gelatin). Ø = 81 ± 34, 66 ± 30, 58 ± 27, 50 ± 19, 42 ±17, and 20 ± 6 nm for 0.2, 0.3, 0.4, 0.6, 1.0, and 3.0 µM [HAuCl_4_], respectively; n = 150. |

| 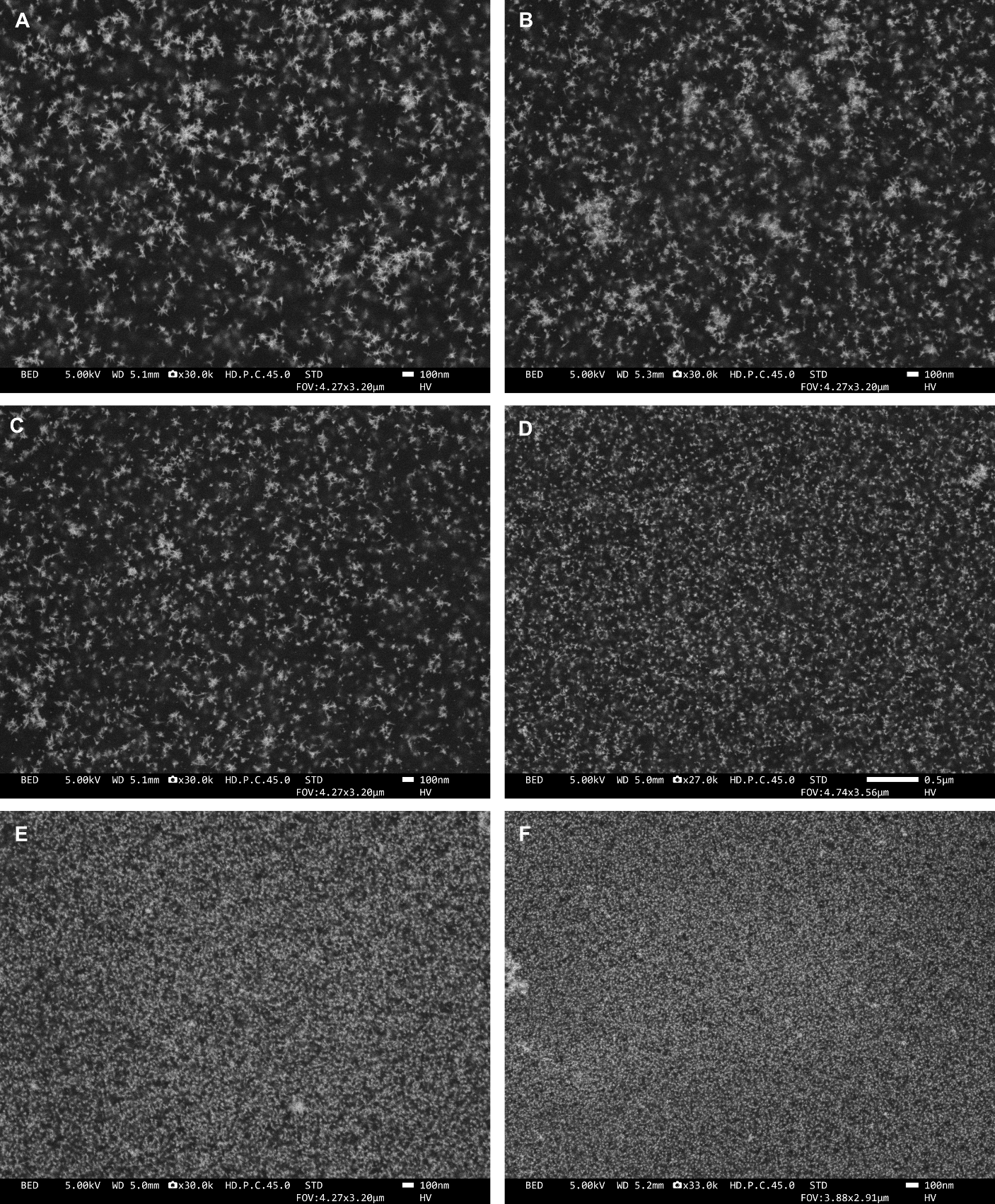 |
| --- |
| **Figure S8. A-F:** Additional SEM images of the nanoparticles on dried gelatin hydrogels (10%) obtained under different seeding conditions: (**A**) 0.2, (**B**) 0.3, (**C**) 0.4, (**D**) 0.6, (**E**) 1.0, and (**F**) 3.0 µM [HAuCl_4_], respectively. |

We analyzed the statistical information from SEM images in detail for the different samples (**Figure S9**). The quantity of nanoparticles with the indicated branch numbers in **Figure S9E** are represented as percentages from the total number of analyzed particles to facilitate sample-to-sample comparison.


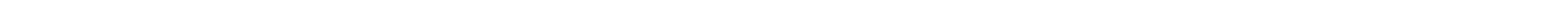
**
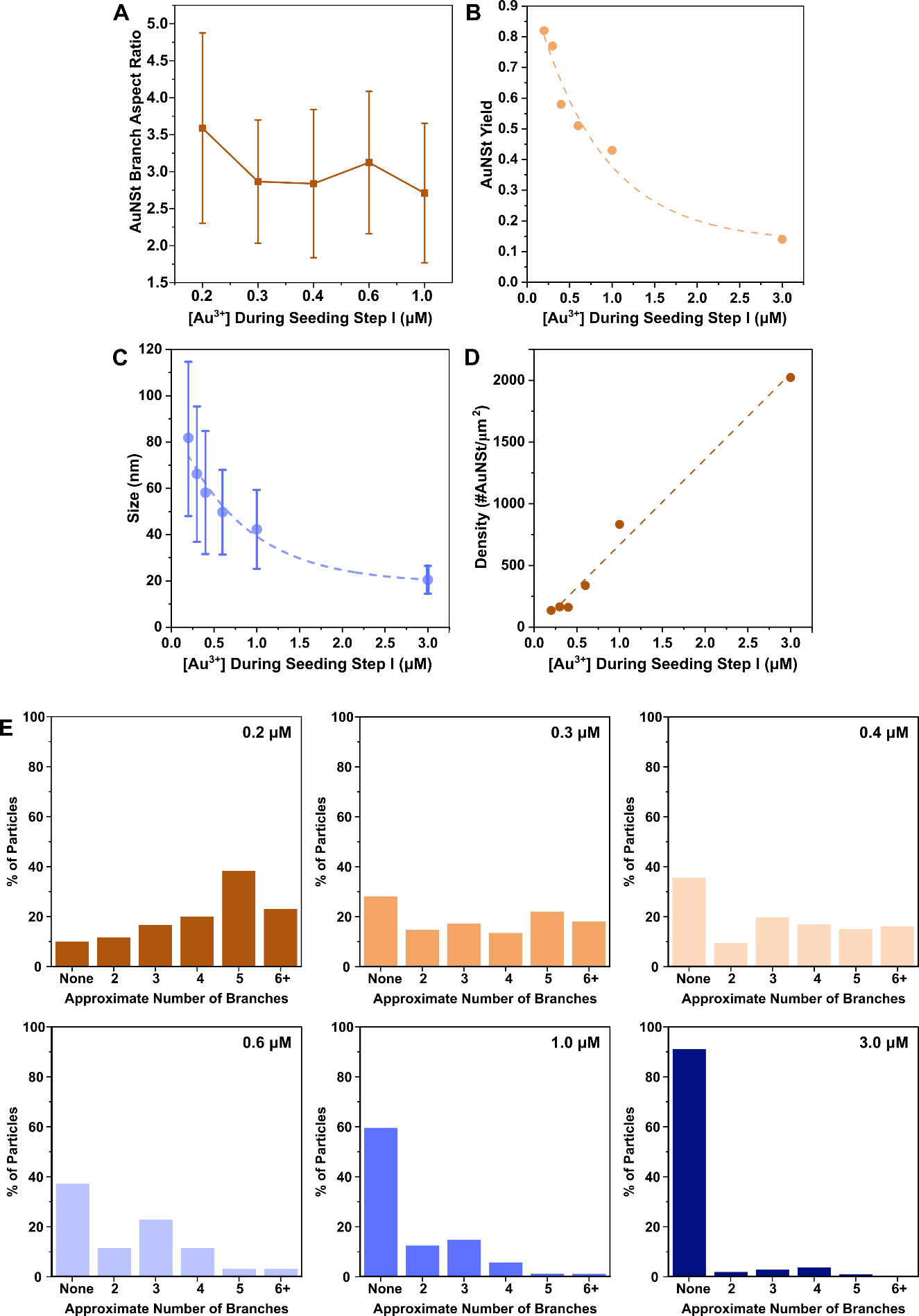
**

**Figure S9. A:** Average branch aspect ratio at different seeding conditions for **Step I** (n = 50 branches)**. B:** Measured yield of branched products for each sample (n = 150). **C:** Average size of the particles obtained each condition (n = 150). **D:** Density of particles on the surface (n = 150). **E:** Approximate percentage of nanoparticles with indicated number of branches. The actual number of analyzed particles depended on the SEM image, all nanoparticles in a given region were analyzed to reduce bias during the measurements taken by hand. Therefore, the N values for the 0.2, 0.3, 0.4, 0.6, 1.0, and 3.0 µM samples were: 60, 82, 107, 97, and 112, respectively.

*Note: The measured branch aspect ratios and branch numbers are estimations, and slightly underestimate the actual values, because in 3D the branches may be oriented away or towards the detector and only their projection (not their actual length) can be measured.*

Under the seeding conditions shown for **Figure 2** in the **Main Text** and in **Figures S7-S9**, the seeds were too small to be observed by TEM and hardly any extinction could be measured with UV-vis spectroscopy after seeding in all cases. However, increasing the gold precursor concentration to 0.1 mM or higher did yield a color change that was measurable and visible to the naked eye (**Figure S10A,B**). When 1.2 mM gold precursor was used (60× the amount used in the seeding conditions for AuNSt), the seeds could even be measured with SEM (**Figure S10C,D**).

| 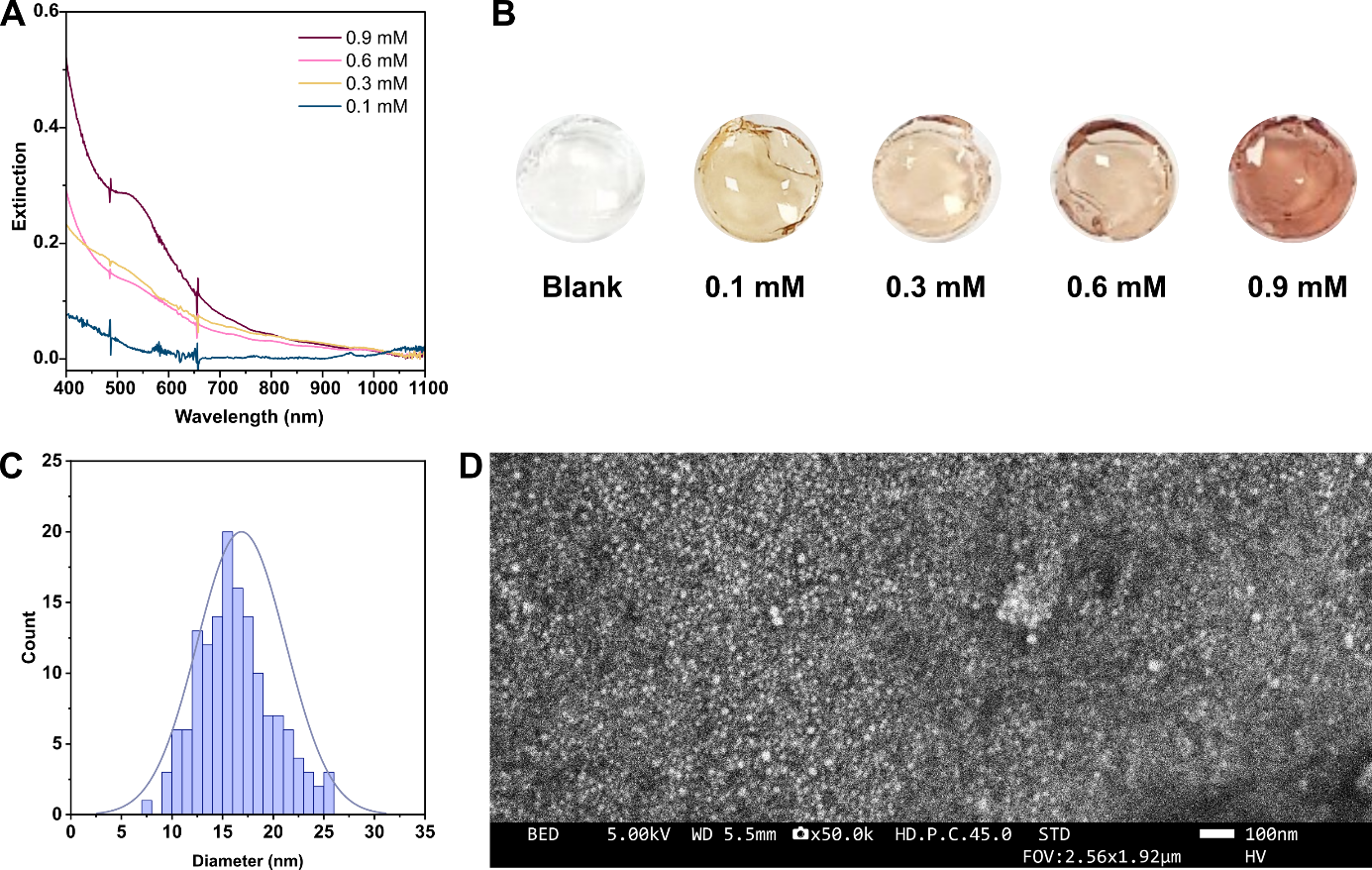 |
| --- |
| **Figure S10.** Synthesis of “large seeds”: **A:** UV-vis extinction spectra of large seeds on gelatin 10% w/v. **B:** Corresponding digital photographs of the products. **C:** Size distribution of seeds obtained at 1.2 mM HAuCl_4_ during the seeding step **(**Ø = 17 ± 4 nm; n = 150). **D:** Corresponding SEM image of large seeds. |
| **B. Additional spectroscopic characterization of gold ion interactions with hydrogels** |
| One and two-dimensional ^1^H-NMR were used to identify key amino acid residues in gelatin, so that the gold binding to those residues could be analyzed (**Figure 3 Main Text**, **Figure S11**). |

| 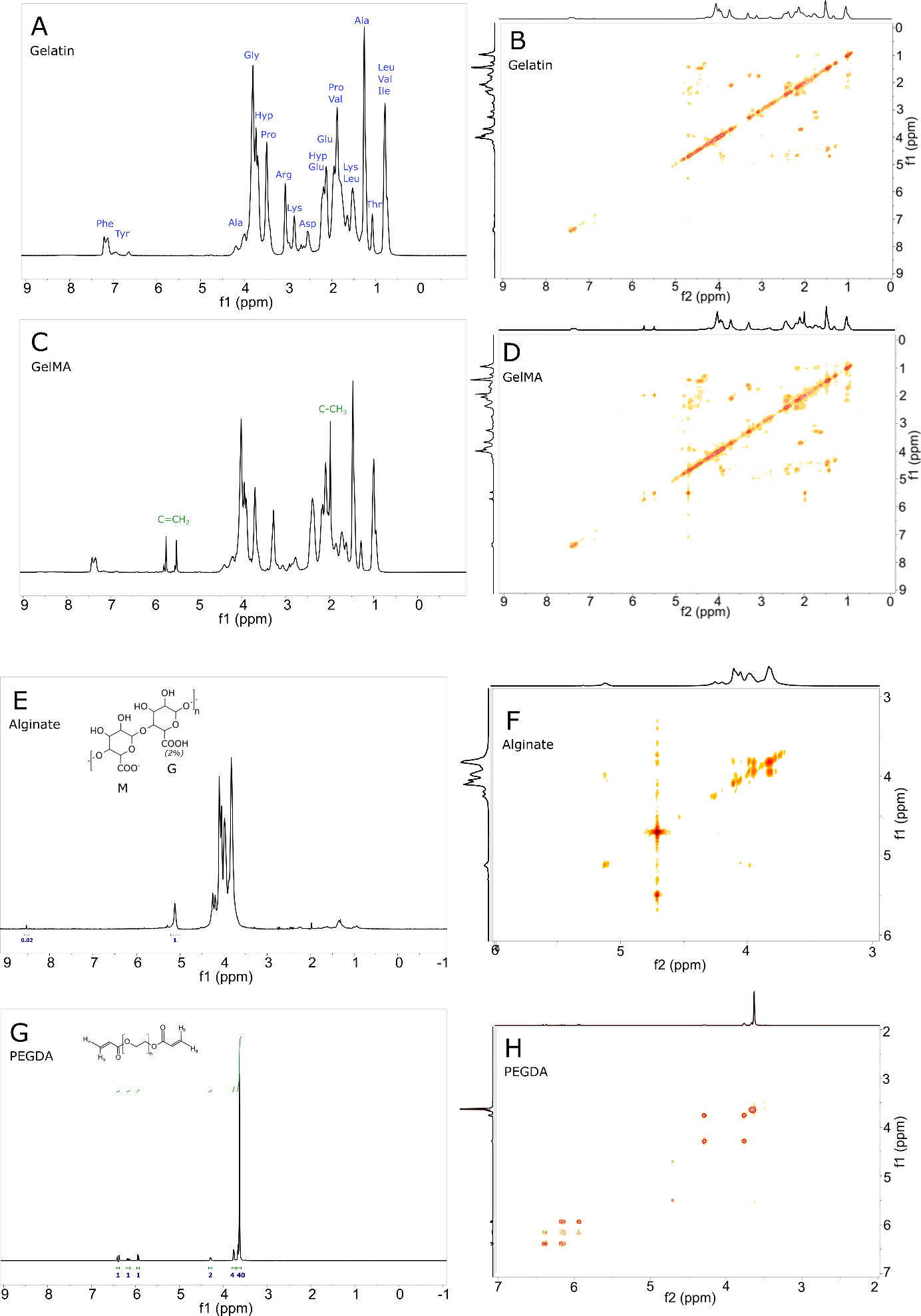 |
| --- |
| **Figure S11.** Full ^1^H NMR spectra, along with a 2-dimensional COSY experiments, were used for complete characterization and proton assignments for: **A:** Gelatin: ^1^H NMR (500 MHz, D_2_O) δ (ppm) 7.52 – 7.26 (Phe), 7.25 – 7.04 (Tyr), 6.86 (Tyr), 4.52 – 4.18 (Ala), 4.15 – 3.82 (Gly, Hyp), 3.67 (Pro), 3.28 (Arg), 3.08 (Lys), 2.76 (Asp), 2.38 (Gly, Hyp), 2.25 – 1.91 (Glu, Pro, Val), 1.82 – 1.61 (Lys, Leu), 1.47 (Ala), 1.29 (Thr), 1.11 – 0.85 (Leu, Val, Ile). **B:** ^1^H-^1^H COSY spectrum of gelatin. **C:** GelMA: ^1^H NMR (500 MHz, D_2_O) δ (ppm) 7.50 – 7.27 (Phe), 7.16 (Tyr), 6.87 (Tyr), 5.76 (C=CH_2_), 5.51 (C=CH_2_), 4.51 – 4.20 (Ala), 4.16 – 3.83 (Gly, Hyp), 3.78 – 3.54 (Pro), 3.39 – 3.17 (Arg), 2.97 – 2.70 (Lys), 2.56 – 2.30 (Asp), 2.26 – 2.02 (Gly, Hyp), 1.99 (C-CH_3_), 1.78 – 1.58 (Lys, Leu), 1.47 (Ala), 1.30 (Thr), 1.09 – 0.87 (Leu, Val, Ile). **D:** ^1^H-^1^H COSY spectrum of GelMA. **E:** Alginate: ^1^H NMR (500 MHz, D_2_O) δ (ppm) 8.53 (COOH), 5.11 (O-CH), 4.32 – 4.16 (CH-COO), 4.14 – 4.02 (CH-CH-COO), 3.97 (CH-OH), 3.91 – 3.69 (CH-OH). **F:** ^1^H-^1^H COSY spectrum of Alginate. **G:** PEGDA: ^1^H NMR (500 MHz, D_2_O) δ (ppm) 6.39 (d, J = 18.5 Hz, 1H, CH_2_ =CH, H_a_), 6.16 (dd, J = 17.8, 10.7 Hz, 1H, CH=CH_2_), 5.94 (d, J = 11.3 Hz, 1H, CH_2_ =CH, H_b_), 4.33 – 4.25 (m, 2H, CO-OCH_2_), 3.76 (dd, J = 5.6, 3.5 Hz, 2H, CO-OCH_2_CH_2_), 3.63 (s, 40H, OCH_2_CH_2_). **H:** ^1^H-^1^H COSY spectrum of PEGDA. |

FTIR was also used to characterize gold binding to hydrogels with different formulations (**Figures 3,5,6** in the **Main Text** and **Figure S12**).

| 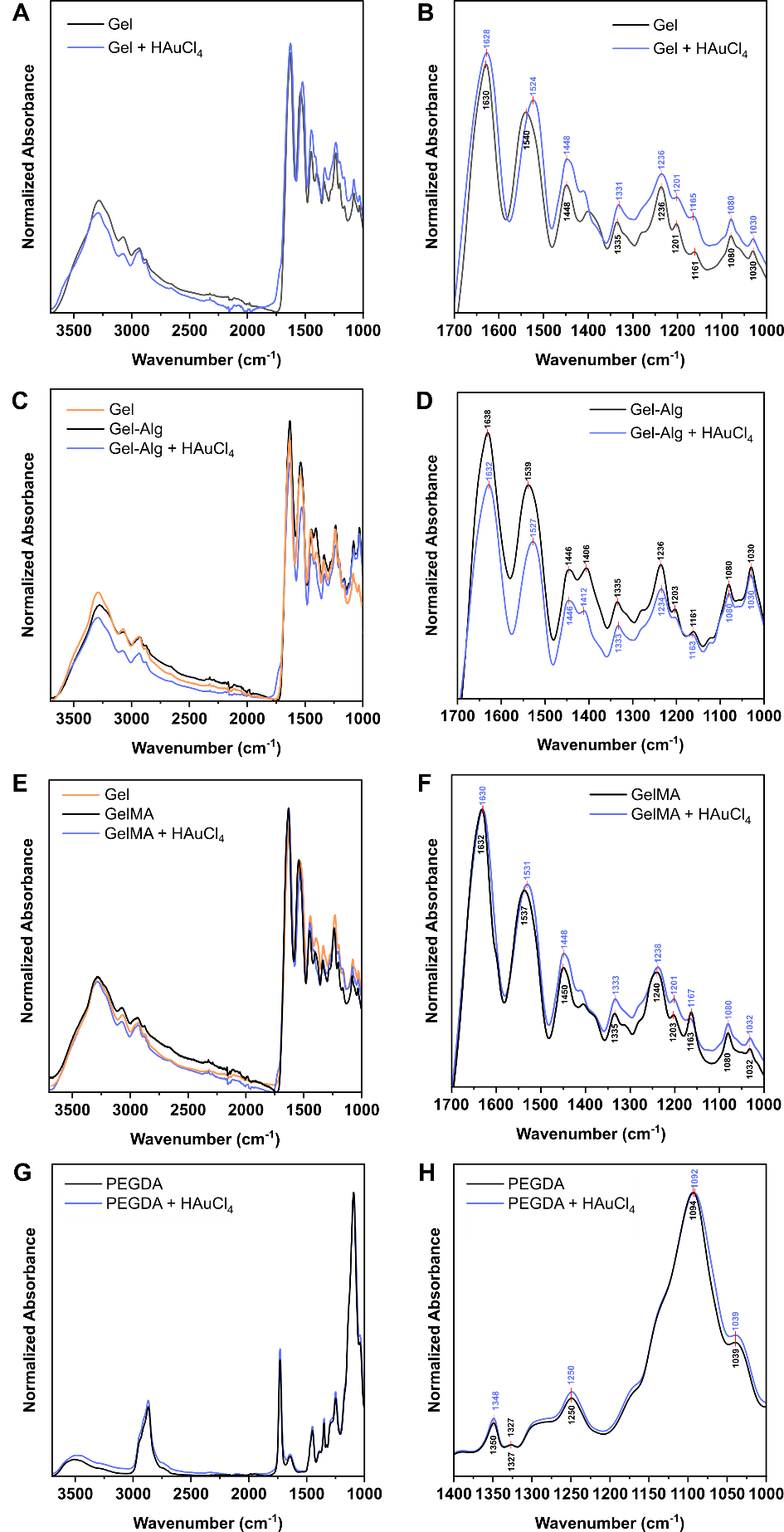 |
| --- |
| **Figure S12. A-H:** FTIR spectra of the different hydrogel formulations with and without incubation in 50 mM HAuCl_4_. |

**C. Rheological and swelling properties of hydrogels**

The rheological properties of the prepared hydrogels were evaluated by performing an amplitude sweep to identify the linear viscoelastic region (LVE) where non-destructive deformation of the gel occurs. From the amplitude sweep, it can be observed that PEGDA is rather brittle compared to the other gels because the yield point (τ_y_) and flow point (τ_f_) are nearly equal. Frequency sweeps were then performed selecting a sheer strain value within the LVE from the amplitude sweeps (**Figure S13 E-H**). In all gels, storage modulus (G’) > loss modulus (G”). The greater contribution of the elastic component of the viscoelastic behavior indicates the successful formation of hydrogels in all cases.

| 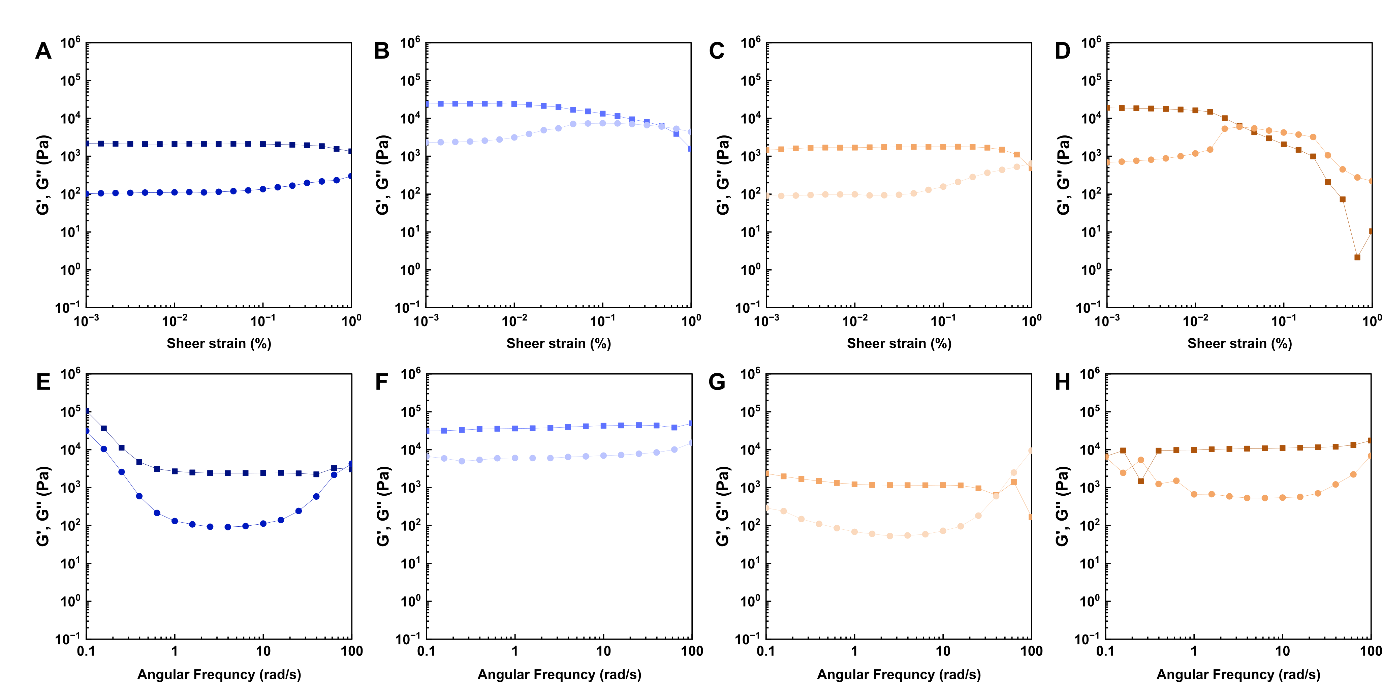 |
| --- |
| **Figure S13. A-D:** Amplitude sweeps to determine the linear viscoelastic (LVE) region of the different hydrogels: (**A**) gelatin 10% w/v, (**B**) gelatin 10% -alg 2% w/v, (**C**) gelatin methacryloyl 10% w/v, (**D**) poly(ethylene glycol) diacrylate 10% v/v. **E-H:** Representative frequency sweeps showing G’ and G”, the storage and loss moduli, respectively for the corresponding formulations. G” is denoted by the darker color in all graphs, and is higher than G’ in all cases, indicating that all the formulations formed hydrogels. |



G’ and G” describe the capability of the material to deform and flow (*e.g.,* a higher G’ value indicates a higher stiffness). Of the four different materials, the G’ and G” values followed the trend from lowest to highest: GelMA 10% *w/v*, gelatin 10 % *w/v*, PEGDA 10% *v/v*, and Gel 10% -Alg 2% *w/v* (**Figure S14**).

| 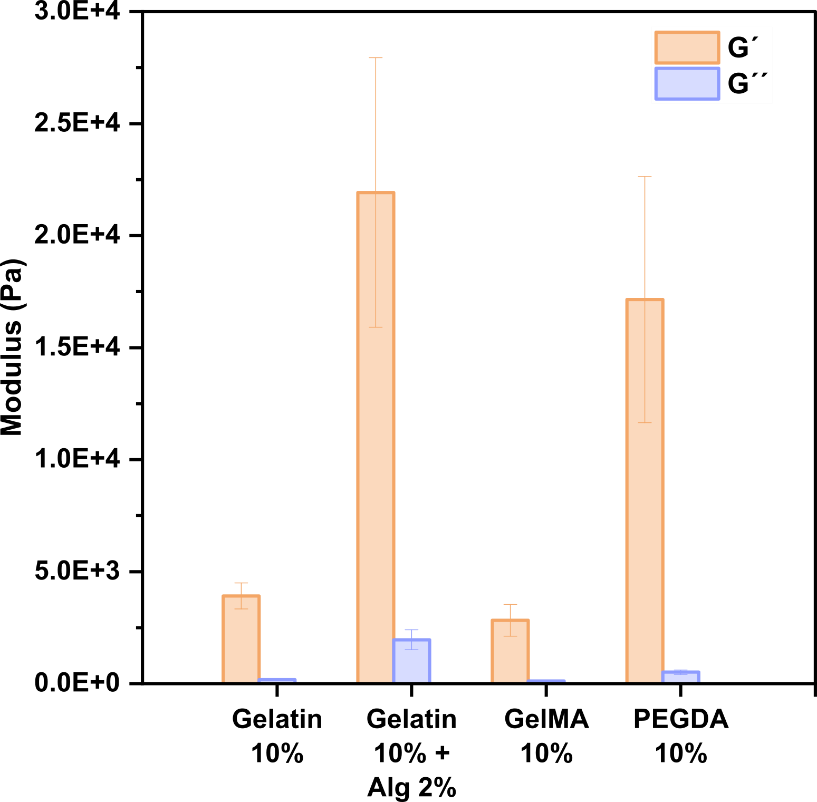 |
| --- |
| **Figure S14.** Comparison of the G’ and G” values for the different formulations across 3 measurements. |

The swelling of the as-prepared hydrogels was evaluated both in AuNSt growth solution and in water (**Figure S15**).

| 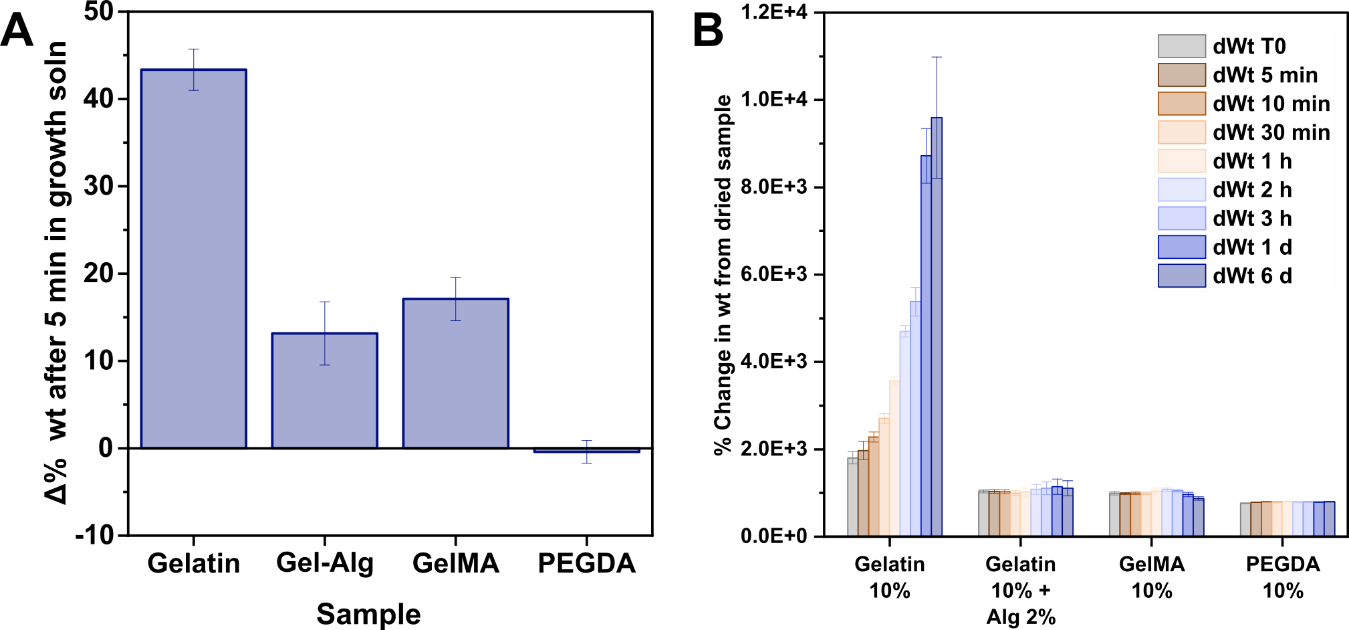 |
| --- |
| **Figure S15. A:** Percent change in weight of the as-prepared hydrogels after incubation in AuNSt growth solution for 5 min. **B:** Swelling of the as-prepared hydrogels in water. All measurements were performed in triplicate. |

**D. UV-vis spectroscopy gold binding experiment**

Gold ion binding to the hydrogels was evaluated by UV-vis spectroscopy as described in **Figure 3** in the **Main Text**. The full UV-vis spectra at the different timepoints of incubation are shown in **Figure S16**. The HAuCl_4_ solution contains both AuCl_4_^−^ as well as hydroxylated species (*i.e.*, AuCl_3_(OH)^−^), which have ligand-metal transition bands at ~218 and ~288 nm (**Figure S16**).^9^ As the hydrogel is incubated in the gold salt solution, the intensity of both peaks decreases with time as expected. A series of control experiments incubating the different hydrogels in only MilliQ water were also completed. The absorbance in the UV around 190 nm increased for all gels after 60 min, which may be due to leaching of organic materials from the hydrogels (**Figure S16**). For GelMA and PEGDA, UV-absorbing photoinitiators are required to initiate the reactions that yield covalent binding in the hydrogels. The UV-vis spectra of two common photoinitiators, 2-hydroxy-4′-(2-hydroxyethoxy)-2-methylpropiophenone (irgacure, Ig) and lithium phenyl (2,4,6-trimethylbenzoyl) phosphinate (LAP) absorbed in the UV range, with Ig giving two major peaks overlapping with the gold species of interest (**Figure S16**). On the other hand, although LAP has an absorbance at 370 nm, this peak had relatively low intensity. Therefore, only GelMA and PEGDA prepared with LAP were used to compare in the gold binding experiments. Moreover, only the change in intensity of the peak at ~218 nm over time was monitored because of its higher intensity. Overall, the trend in gold sequestration determined *via* UV-vis spectroscopy from most to least absorbed gold was: gelatin 20% *w/v*, gelatin 10% *w/v*, GelMA 10% *w/v*, gelatin 5% *w/v*, Gel 10% -Alg 2% *w/v*, and PEGDA 10% *v/v*.

| 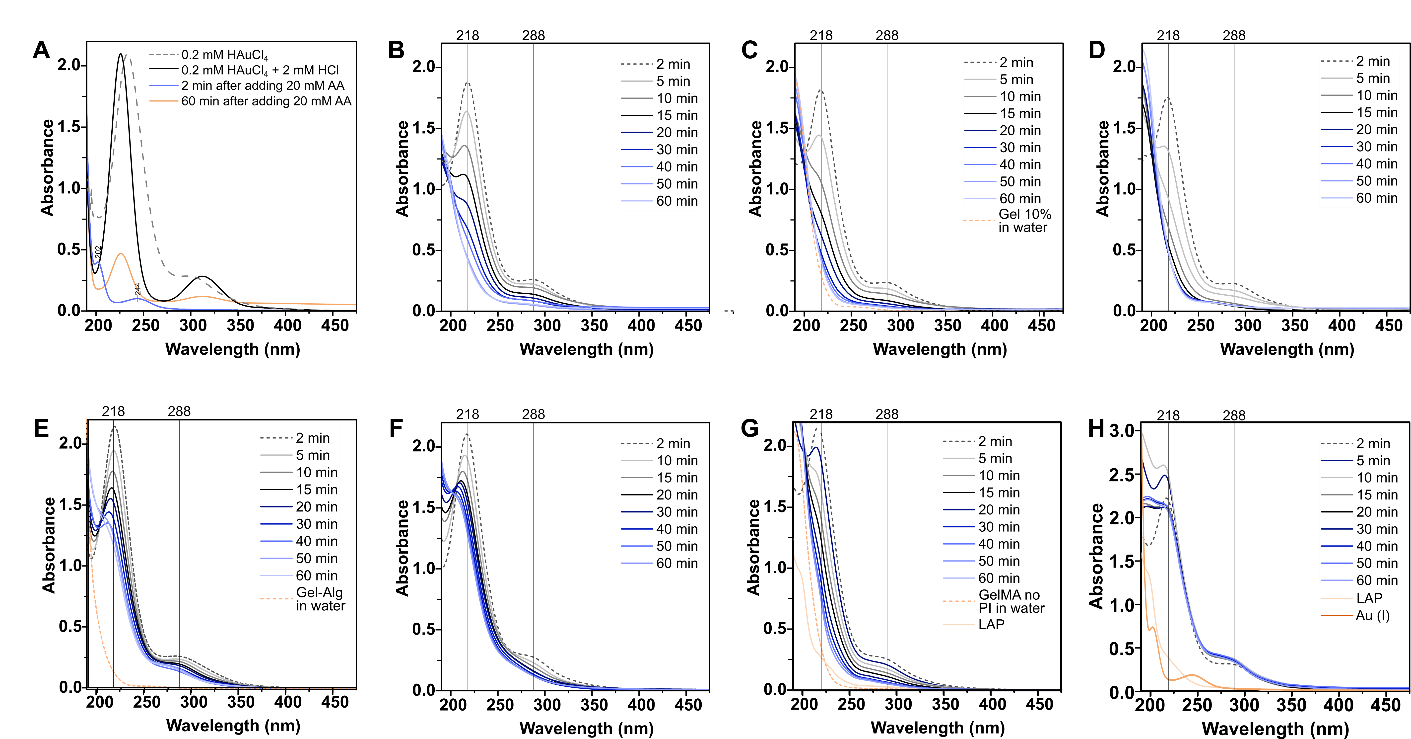 |
| --- |
| **Figure S16. A:** UV-vis absorbance spectra for the experiment evaluating the contribution of Au^1^ to the absorbance of the gold solutions (l = 0.5 cm). **B-H:** UV-vis spectra for the gold binding experiments for (**B**) gelatin 5% w/v, (**C**) gelatin 10% w/v, (**D**) gelatin 20 % w/v, (**E**) gelatin 10% -alginate 2% w/v cured in 100 mM CaCl_2_ for 5 min, (**F**) uncured gelatin 10% - alginate 2% w/v, (**G**) gelatin methacryloyl (GelMA) 10% w/v cured with 0.15% w/v lithium phenyl-2,4,6-trimethylbenzoylphosphinate (LAP), (**H**) poly(ethylene glycol) diacrylate (PEGDA) 10% v/v cured with 0.15% LAP. |

**E. AuNSt synthesis on PEGDA gels**

PEGDA does not produce AuNSt with high yield, which was expected considering that both FITR and NMR show little to no shifts when the polymer is incubated with gold precursors (**Figure S17A-C**). Indeed, SEM confirms the poor yield of AuNSt on PEGDA (**Figure S17D**).

| 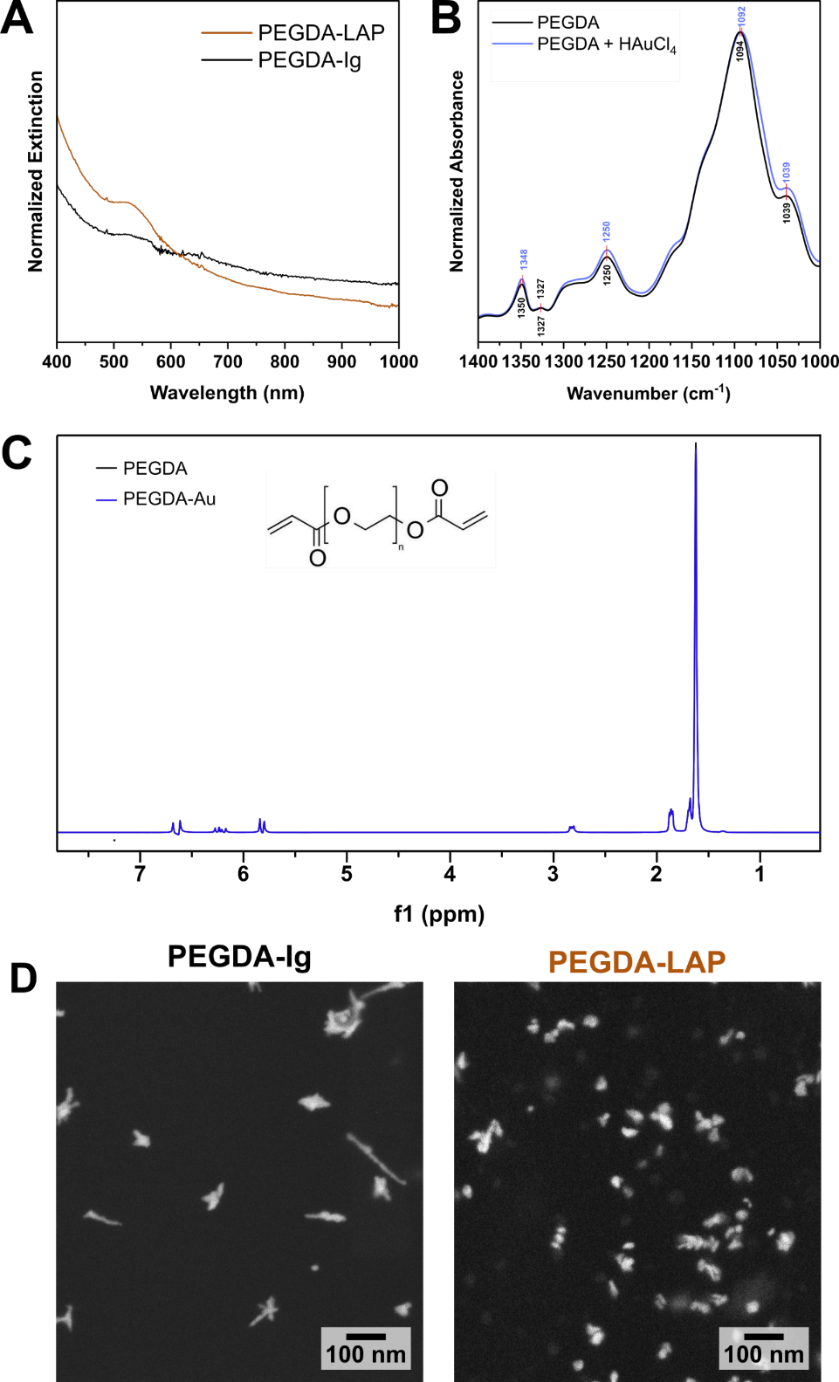 |
| --- |
| **Figure S17. A:** UV-vis spectra of the products obtained with poly(ethylene glycol) diacrylate (PEGDA) using different photoinitiators. **B:** FTIR spectra of freeze dried PEGDA hydrogels without and with incubation in 50 mM HAuCl_4_ solution for 5 min. **C:** ^1^H-NMR spectra of PEGDA without and with the addition of 4 µL/mL 50 mM HAuCl_4_. **D,E:** SEM images of the products obtained on PEGDA hydrogels with different photoinitiators. |

**F. Additional data for the optimization of AuNSt growth on hydrogels with different polymer content and composition**

When AuNSt grown on gelatin 5% *w/v* were collected for TEM, a population of isotropic products was also observed, which contribute to the extinction at ~530 nm (**Figure S18**). The addition of HCl had different effects, with higher quantities of HCl being more effective for AuNSt growth on gels with higher polymer concentration (**Figure S19**).

| 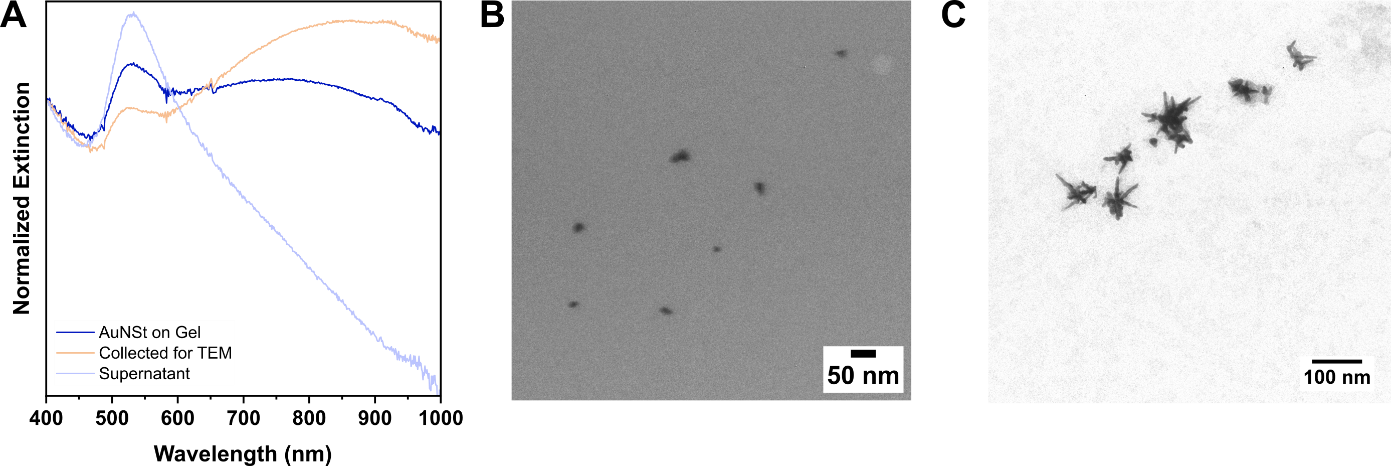 |
| --- |
| **Figure S18. A:** UV-vis spectra of AuNSt grown on gelatin 5% w/v on the hydrogel (dark blue), after hydrogel dissolution and collection of AuNSt (orange), and the corresponding supernatant with a population of isotropic products (light purple). **B-C:** TEM images of the particles observed (**B**) in the supernatant and (**C**) the collected AuNSt. |


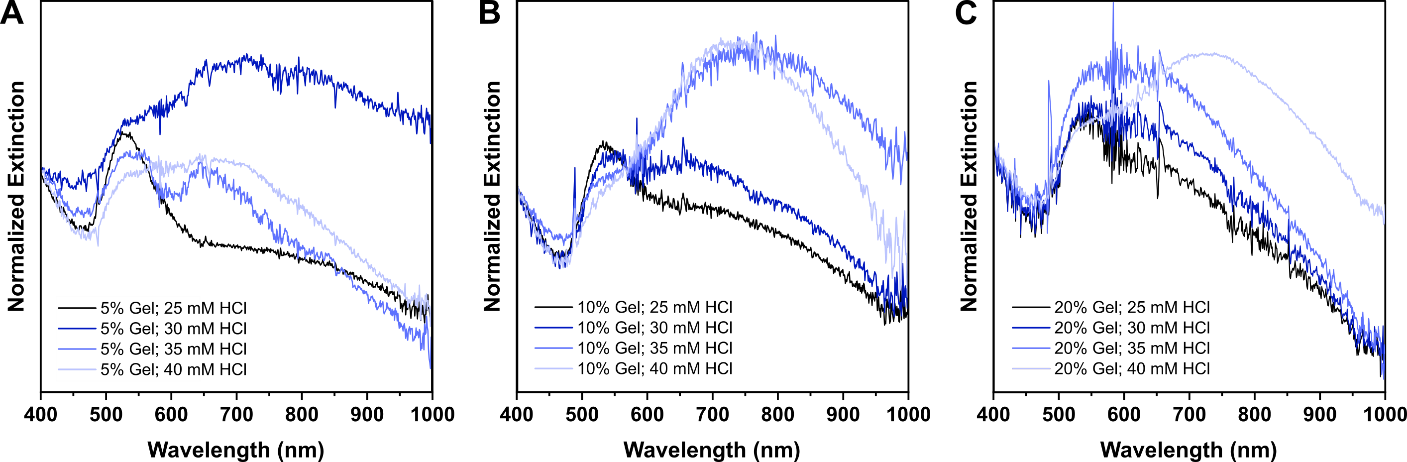


**Figure S19.** UV-vis spectra of products obtained on gelatin at different polymer concentrations: (**A**) 5%, (**B**) 10% and (**C**) 20%, with the addition of different amounts of HCl. Panel **C** repeated here from **Figure 4** in the **Main Text** for comparison.

For gelatin-alginate, the curing of the gel with M^2+^ ions was important to achieve high yields of branched products (Main Text **Figure 5**, **Figure S20**). This can be due to the formation of the “egg box” structure, where functional groups responsible for initiating unwanted side reactions participate in coordinating to the M^2+^ ions (**Figures S21, S22**).

| 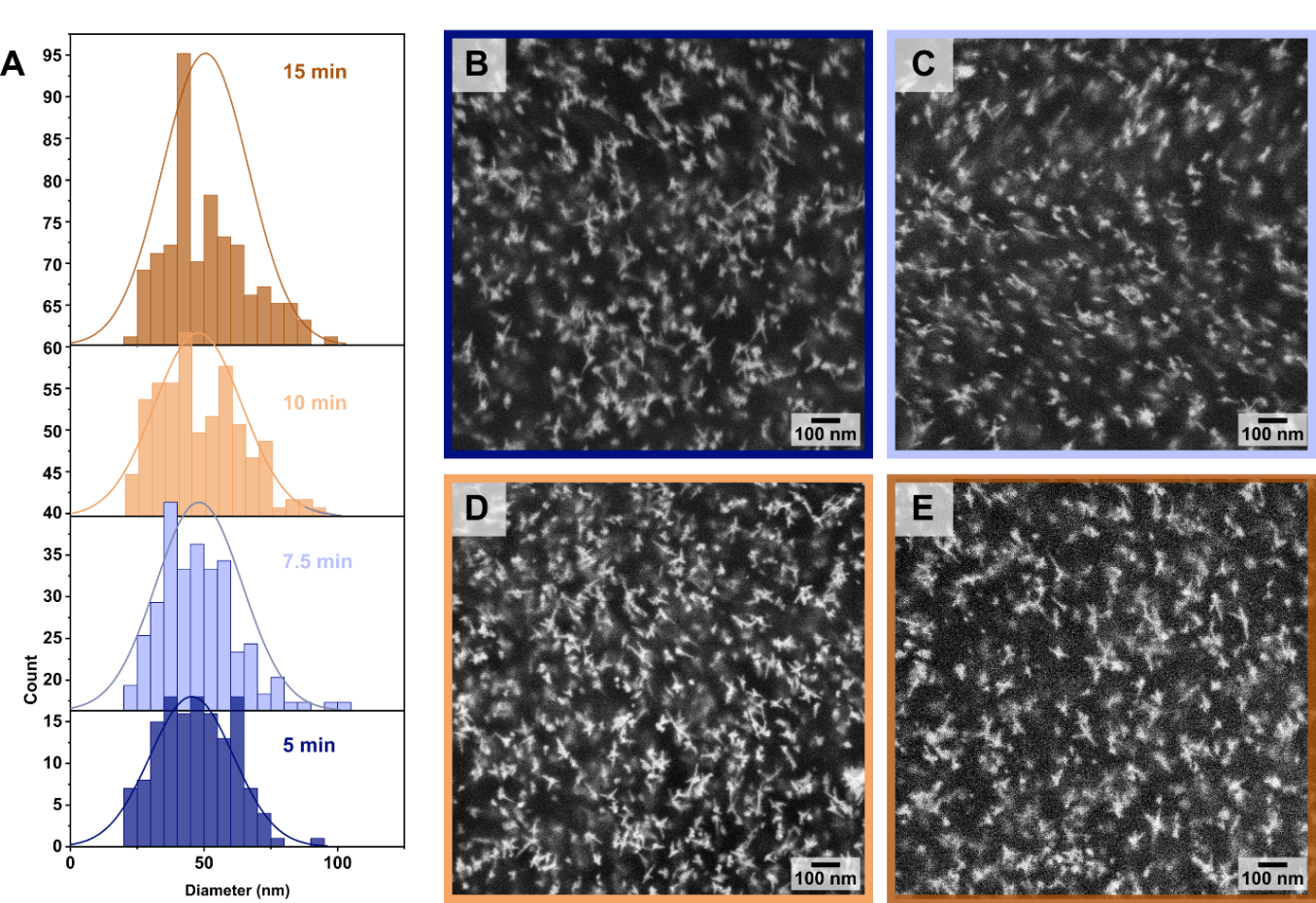  **Figure S20. A:** Size distributions of AuNSt grown on Gel-Alg substrates cured at 5, 7.5, 10, and 15 min. **B-E:** Corresponding SEM images. |
| --- |

| 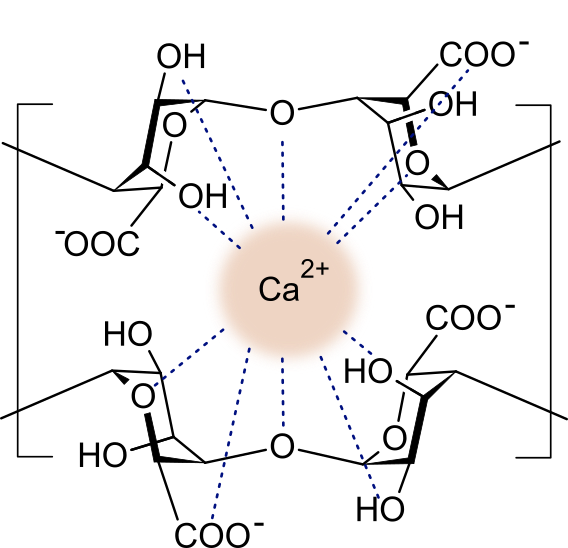 |
| --- |
| **Figure S21. “**Egg box” scheme for alginate curing with M^2+^ ions.^4^ |

| 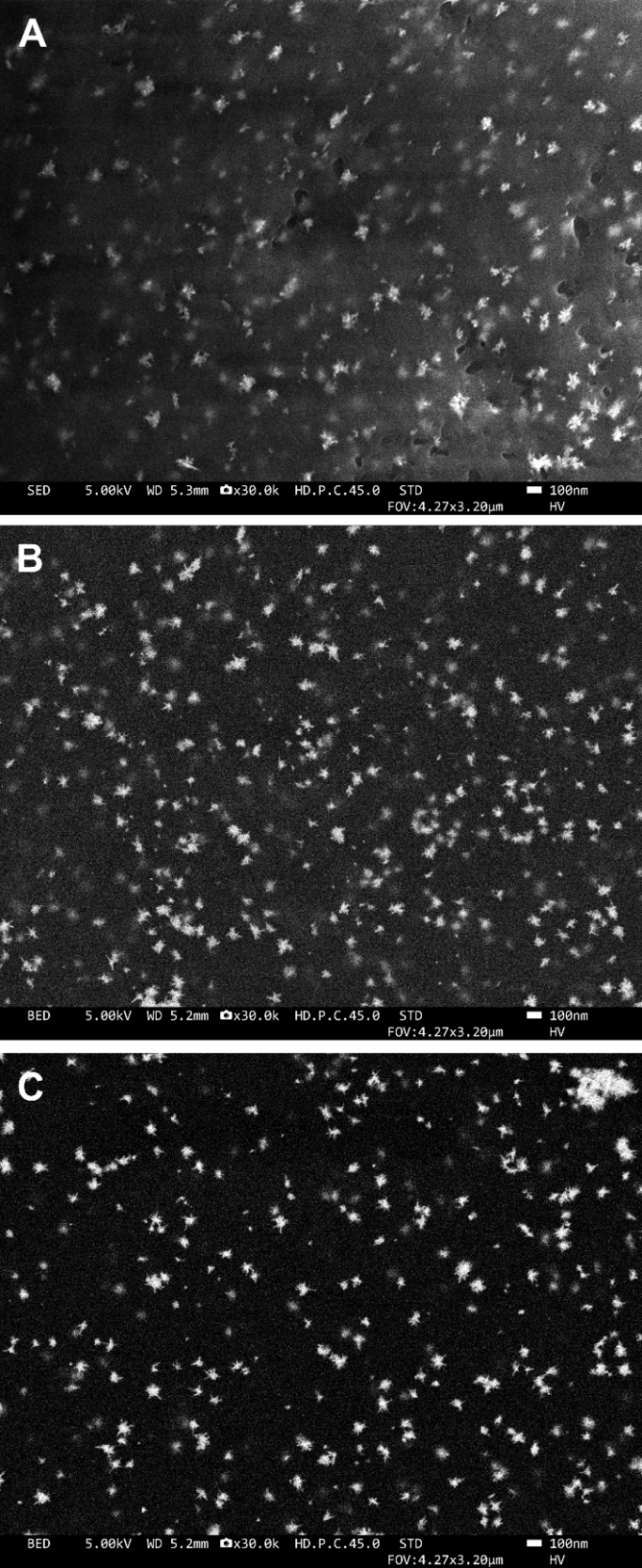 |
| --- |
| **Figure S22. A-C:** Additional SEM images for the products obtained for gelatin-alginate hydrogels after curing in 100 mM CaCl_2_ for (**A**) 30 s, (**B**) 1 min, (**C**) 5 min. |

The yield of methacrylation in the prepared GelMA was evaluated using the approach described in the **Method** **Section II** based on the ^1^H-NMR data shown in **Figure S23**.

| 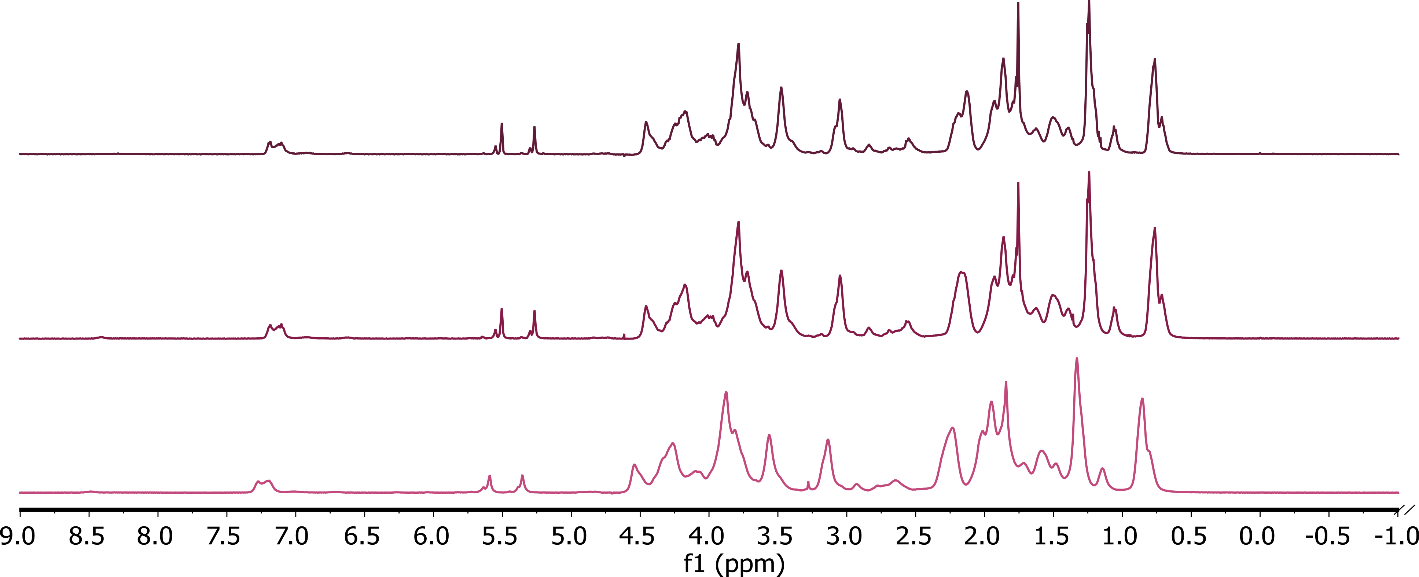 |
| --- |
| **Figure S23. ^1^**H-NMR of different batches of gelatin methacryloyl (GelMA) reported in **Table S1**. |

It was found that taking steps to wash away the excess photoinitiator present in GelMA hydrogels, as well as reducing the HCl concentration assisted in the growth of AuNSt (**Figure 6 Main Text** and **Figure S24**).

| 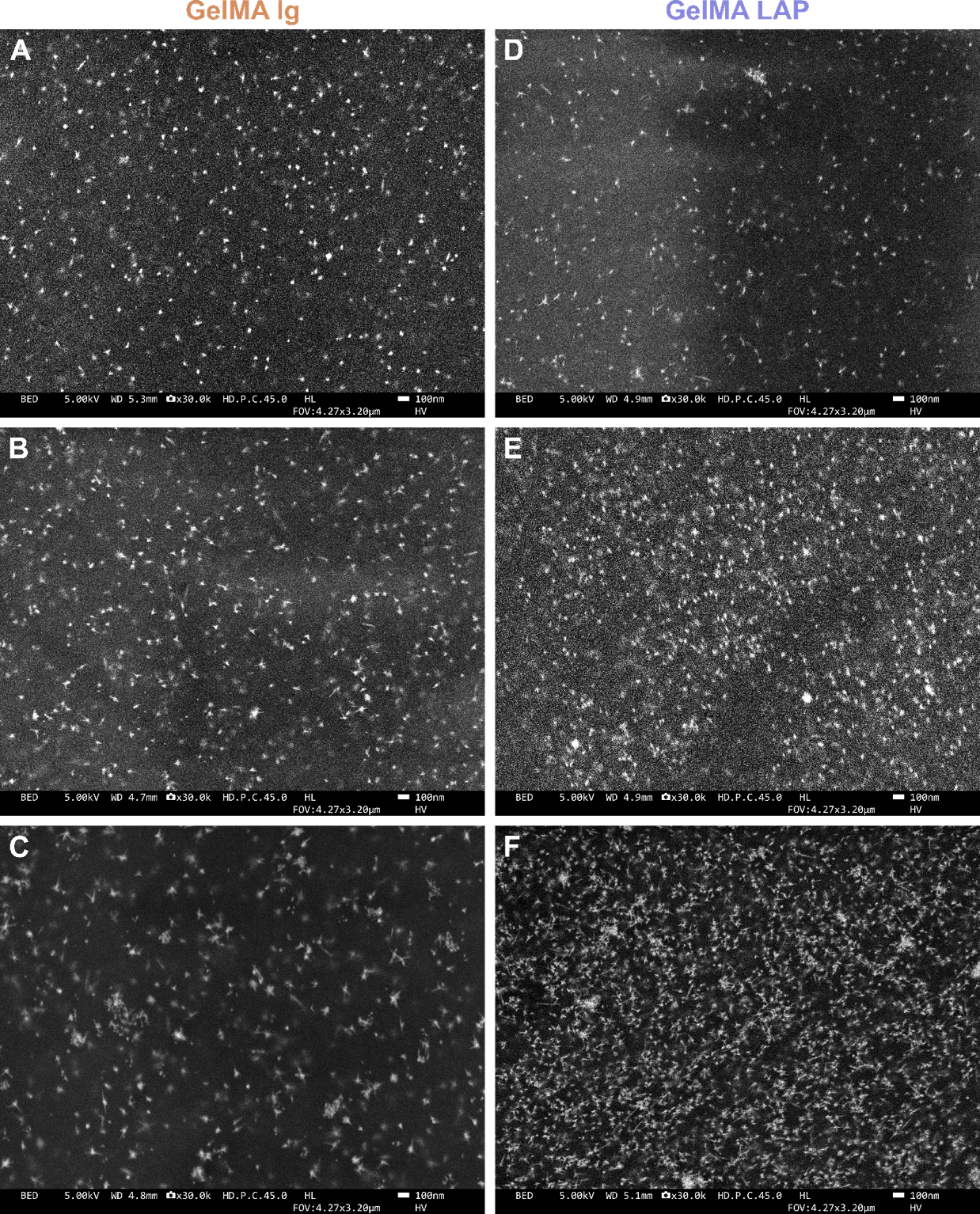 |
| --- |
| **Figure S24. A-C:** Additional SEM images for the products obtained on gelatin methacryloyl (GelMA) hydrogels cured with 2-hydroxy-4′-(2-hydroxyethoxy)-2-methylpropiophenone (irgacure, Ig) (**A**) as-is, (**B**) after washing overnight, and (**C**) after washing overnight and with 25 mM HCl in the growth solution (vs. the standard 30 mM HCl). **D-F:** Additional SEM images for the products obtained on gelatin methacryloyl (GelMA) hydrogels cured with lithium phenyl-2,4,6-trimethylbenzoylphosphinate (LAP) (**D**) as-is, (**E**) after washing overnight, and (**F**) after washing overnight and with 25 mM HCl in the growth solution (vs. the standard 30 mM HCl). |

**G. Additional characterization for the growth of AuNSt in PBS and cell viability studies**

The most branched products giving high extinction in the NIR regime were obtained when no TX was added to the PBS growth solution (**Figure S25**).

| 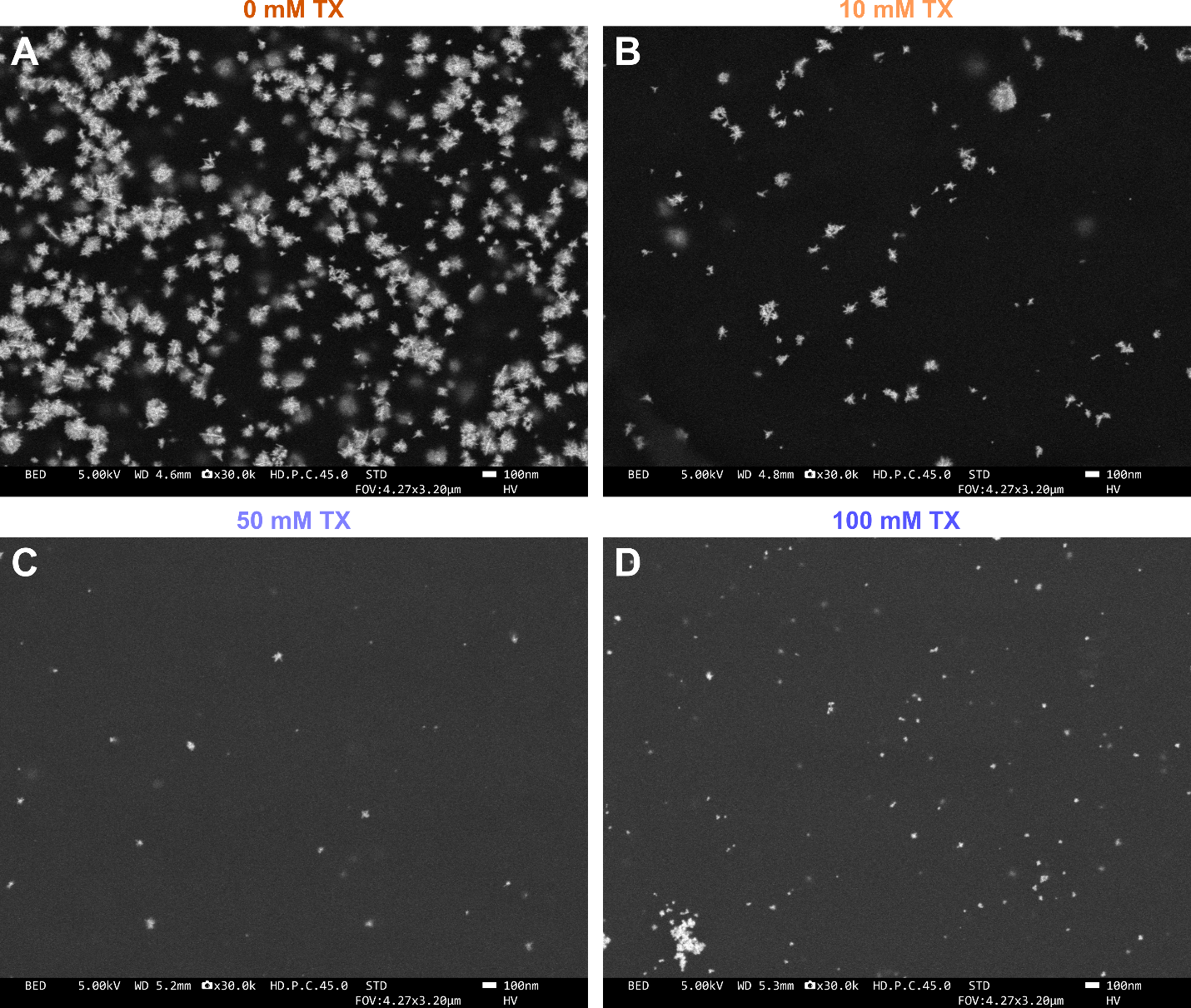 |
| --- |
| **Figure S25.** Additional SEM showing the products obtained from growing AuNSt on gelatin 10% w/v with different concentrations of Triton X-100 (TX). |

The surfactant-free AuNSt could be collected from the 10% *w/v* substrate and analyzed with TEM (**Figures S26,S27**).

| 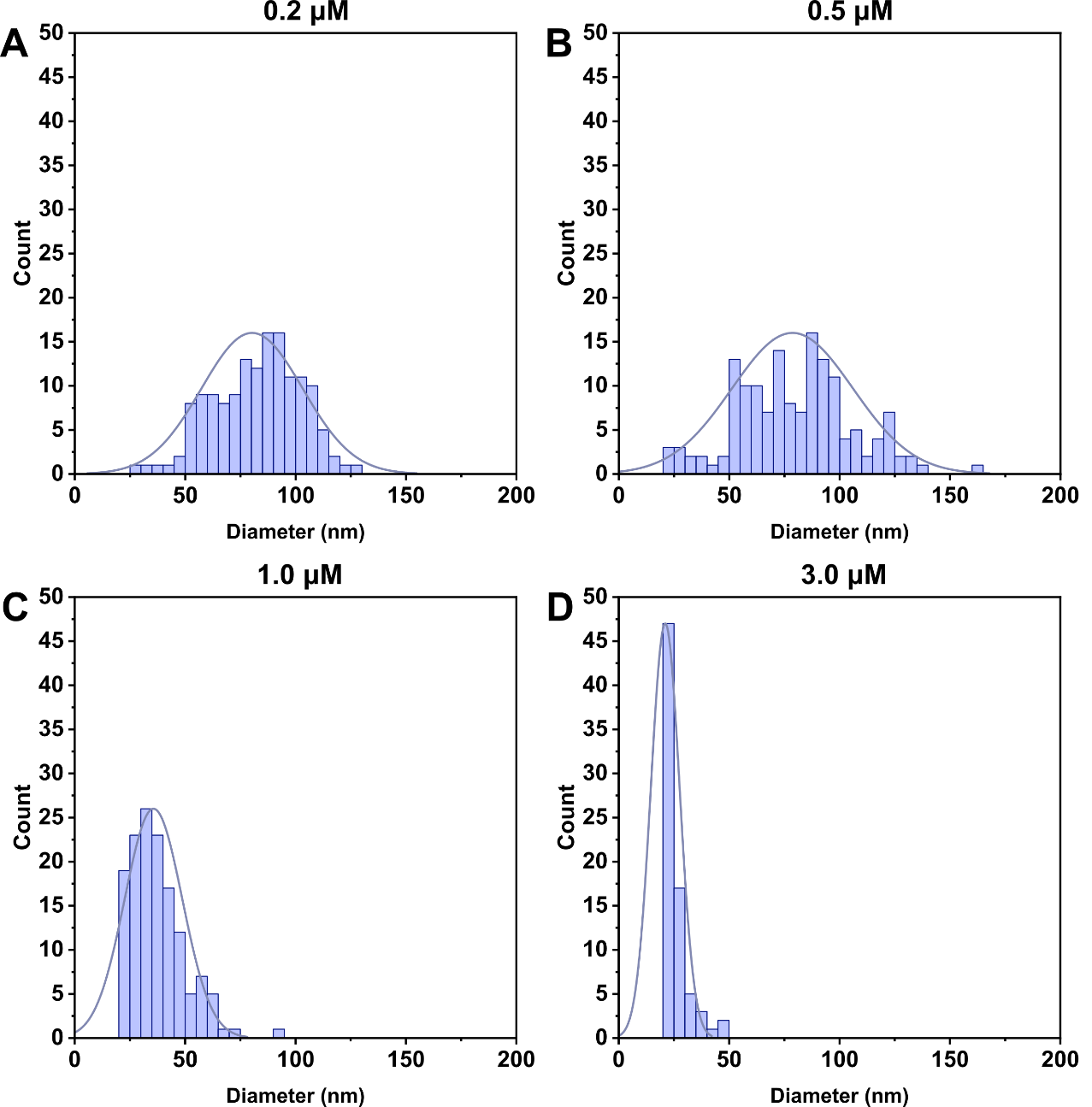 |
| --- |
| **Figure S26. A-D:** Size distributions of Au nanoparticles grown using PBS as the growth medium under different seeding conditions (different HAuCl_4_ concentrations as indicated; n = 150). |

| 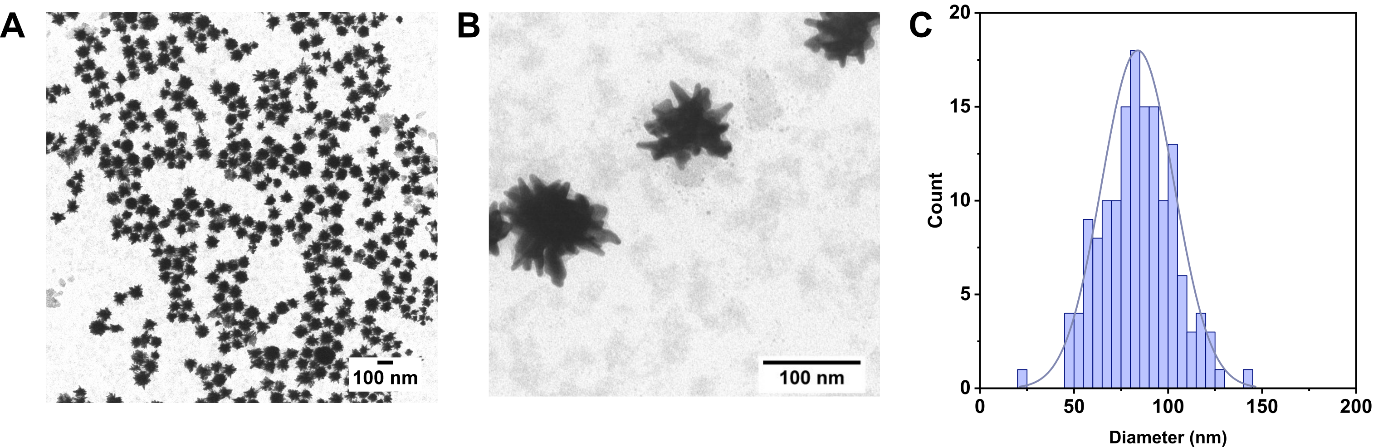 |
| --- |
| **Figure S27. A,B:** TEM images of AuNSt collected from gelatin 10% w/v substrates after growth using PBS as the growth medium (0 mM Triton X-100 and 0.2 µM HAuCl_4_). **C:** Size distribution of the particles; Ø = 84 ± 19 nm, n = 150.  We compared the stability of the surfactant-free PBS AuNSt on day 1 and day 3 of incubation with PBS (**Figure S28**). The UV-vis spectra show similar features, indicating that no significant aggregation or reshaping occurred over this time (**Figure S28 A**). Moreover, comparison of the samples *via* SEM confirm stability, with the products giving the same size distribution on day 1 as on day 3, with no obvious changes in morphology (**Figure S28 B-F**). Therefore, whereas colloidal surfactant-free AuNSt are prone to reshaping and aggregation, we see no evidence of such changes in our case.  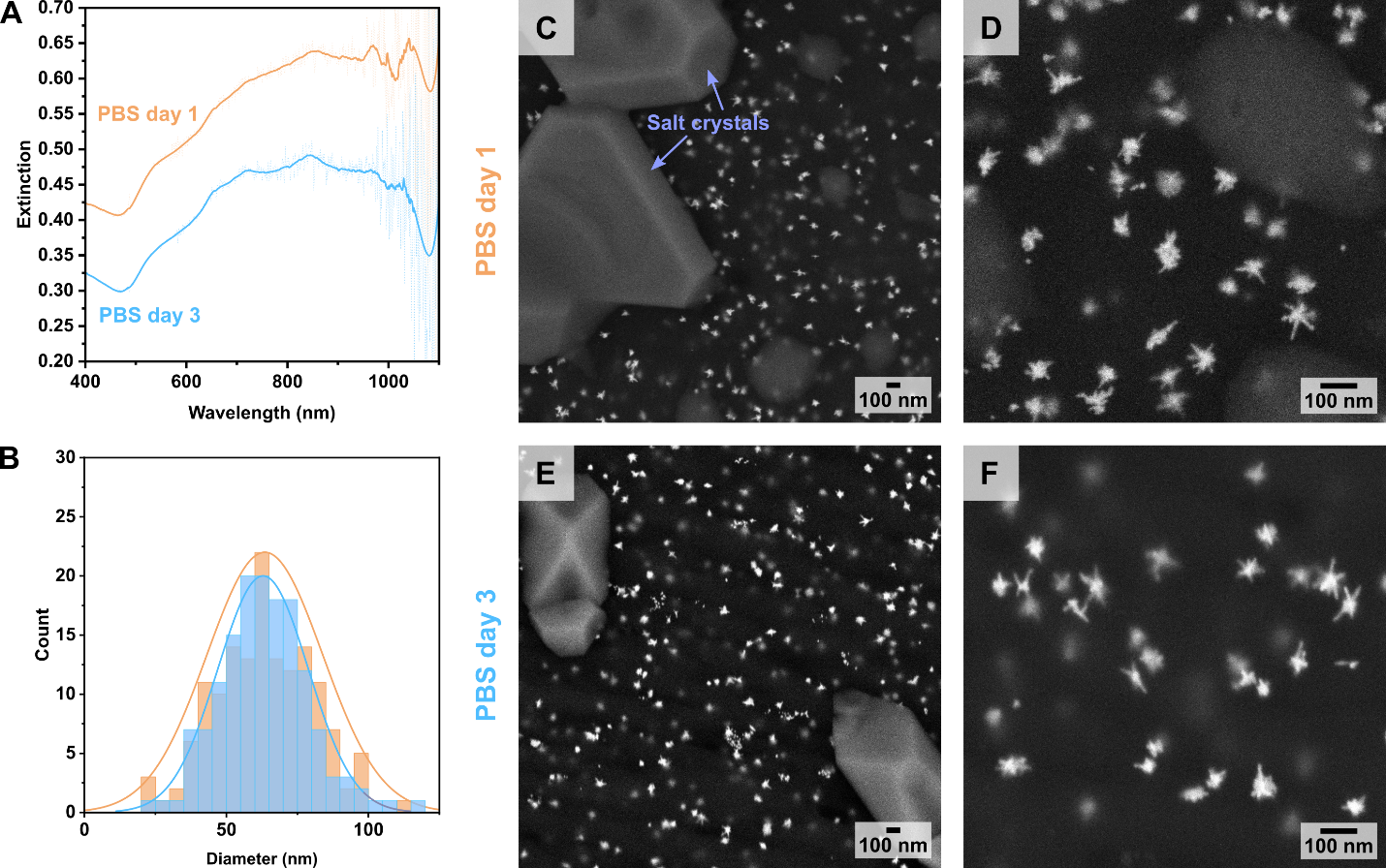  **Figure S28. A:** UV-vis spectra of the AuNSt prepared in PBS on 5% *w/v* gelatin at day 1 and day 3 of incubation in PBS solution. **B:** Measured size distributions of the AuNSt at day 1 (*orange*) and day 3 (*blue*). **C-F:** SEM images of the AuNSt grown in PBS at day 1 (**C,D**) and day 3 (**E,F**). |

The AuNSt morphology was tuned by changing the concentration of gold precursor during **Step I** of the seeding (**Figure S29**).

| 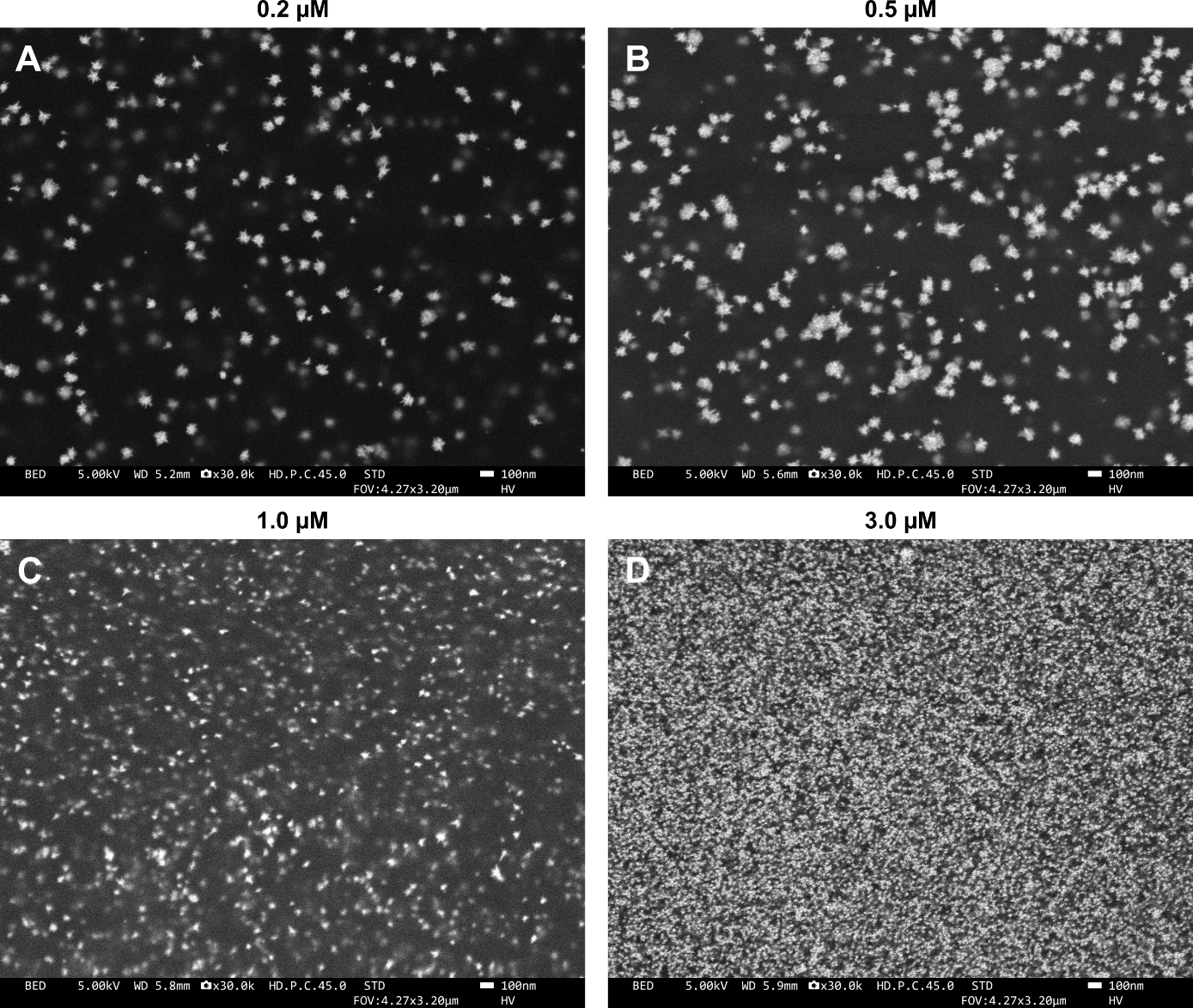 |
| --- |
| **Figure S29.** Additional SEM images showing the products obtained when changing the HAuCl_4_ concentration during **Step I** of the seeding for the synthesis with PBS as the growth medium. |

Surfactant free AuNSt could be synthesized in PBS on both Gel-Alg and gelatin substrates (**Figure S30**).

|  |
| --- |
|  |
| 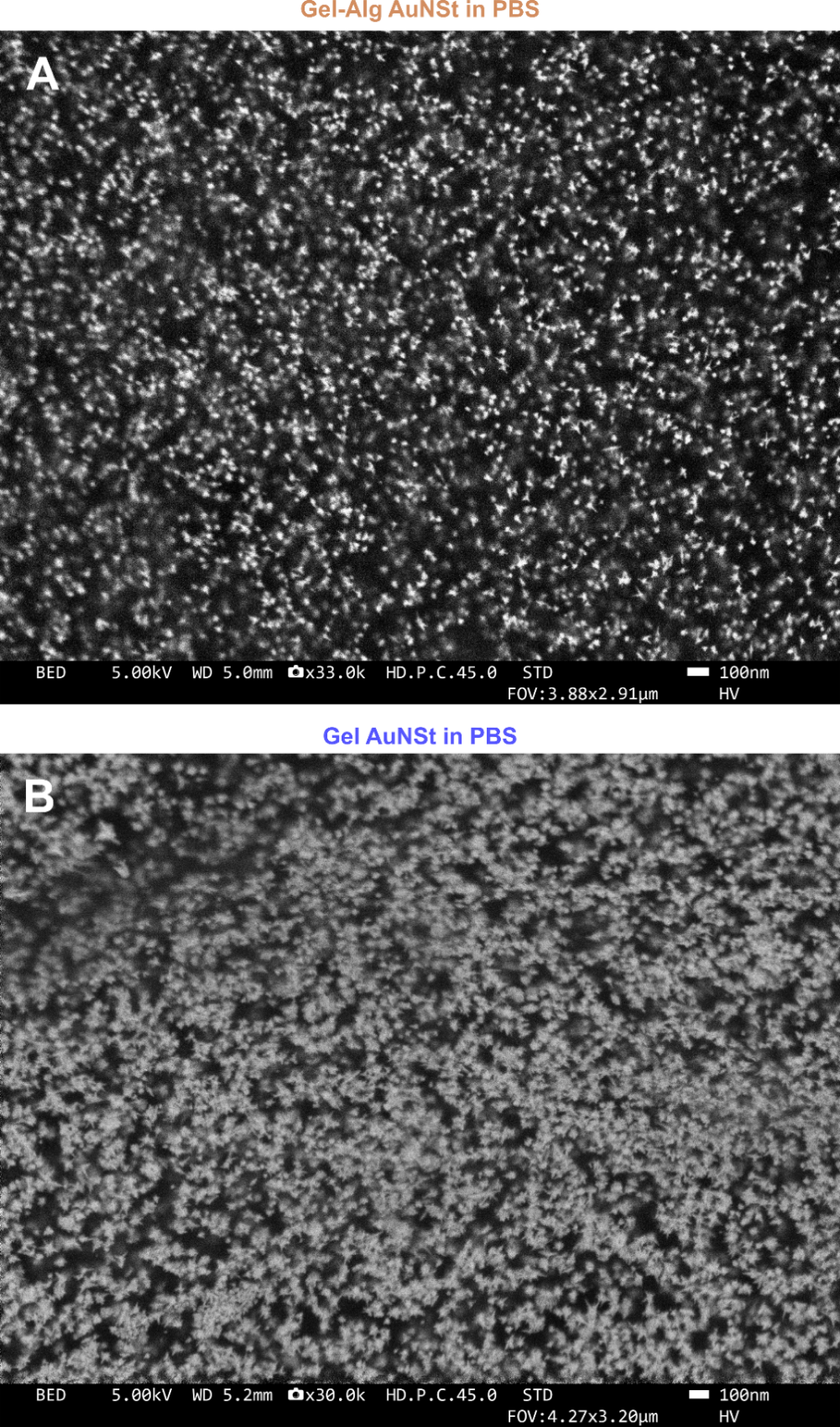 |
|  |
| **Figure S30. A-B:** Additional SEM images showing the AuNSt grown using PBS as the growth medium on (**A**) gelatin 10% - alginate 2% w/v and (**B**) gelatin 10% w/v. |

The biocompatibility of plain hydrogels and those with AuNSt grown with TX in water or surfactant-free in PBS were compared to standard 2D cell culture conditions for MBA-MB-231 breast cancer cells (**Figure 7 Main Text** and **Figure S31**).

| 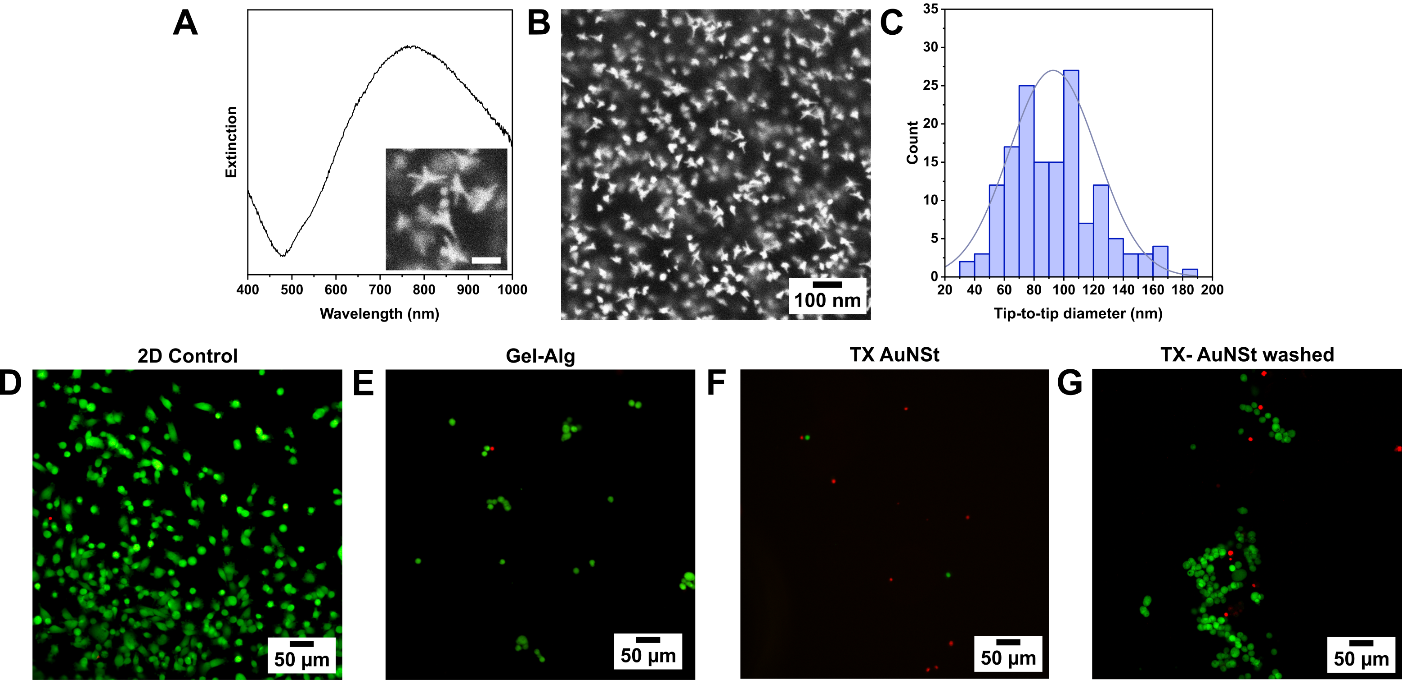 |
| --- |
| **Figure S31. A:** UV-vis extinction spectra of AuNSt grown on gelatin 10% - alginate 2% w/v using PBS as the growth medium (0 mM Triton X-100 and 0.2 µM HAuCl_4_; inset: TEM image of the products). **B:** Additional TEM image of the obtained AuNSt. **C:** Size distribution of the particles; Ø = 93 ± 30 nm; n = 150. **D-G:** Fluorescence microscopy live/dead stain showing the viability of MDA-MB-231 cells cultured for 24 h (**D**) in standard 2D cell culture, (**E**) on plain gelatin-alginate hydrogels, (**F**) on gelatin-alginate hydrogels with Triton X-coated AuNSt synthesized in aqueous growth medium, and (**G**) gelatin-alginate hydrogels with Triton X-coated AuNSt synthesized in aqueous growth medium after washing 5× in (4-(2-hydroxyethyl)-1-piperazineethanesulfonic acid) (HEPES) buffer for 1 h each wash. |

**REFERENCES**

(1) Pallavicini, P.; Donà, A.; Casu, A.; Chirico, G.; Collini, M.; Dacarro, G.; Falqui, A.; Milanese, C.; Sironi, L.; Taglietti, A. Triton X-100 for Three-Plasmon Gold Nanostars with Two Photothermally Active NIR (near IR) and SWIR (Short-Wavelength IR) Channels. *Chem. Commun.* **2013**, *49*, 6265–6267. https://doi.org/10.1039/C3CC42999G.

(2) Atta, S.; Beetz, M.; Fabris, L. Understanding the Role of AgNO_3_ Concentration and Seed Morphology in the Achievement of Tunable Shape Control in Gold Nanostars. *Nanoscale* **2019**, *11*, 2946–2958.

(3) Vinnacombe-Willson, G. A.; García-Astrain, C.; Troncoso-Afonso, L.; Wagner, M.; Langer, J.; González-Callejo, P.; Silvio, D. D.; Liz-Marzán, L. M. Growing Gold Nanostars on 3D Hydrogel Surfaces. *Chem. Mater.* **2024**, *36*, 5192–5203.

(4) Braccini, I.; Pérez, S. Molecular Basis of Ca^2+^-Induced Gelation in Alginates and Pectins: The Egg-Box Model Revisited. *Biomacromolecules* **2001**, *2*, 1089–1096. https://doi.org/10.1021/bm010008g.

(5) Scarabelli, L.; Sánchez-Iglesias, A.; Pérez-Juste, J.; Liz-Marzán, L. M. A “Tips and Tricks” Practical Guide to the Synthesis of Gold Nanorods. *J. Phys. Chem. Lett.* **2015**, *6*, 4270–4279. https://doi.org/10.1021/acs.jpclett.5b02123.

(6) González-Rubio, G.; Llombart, P.; Zhou, J.; Geiss, H.; Peña-Rodríguez, O.; Gai, H.; Ni, B.; Rosenberg, R.; Cölfen, H. Revisiting the Role of Seed Size for the Synthesis of Highly Uniform Sub-10 nm Length Gold Nanorods. *Chem. Mater.* **2024**, *36*, 1982–1997. https://doi.org/10.1021/acs.chemmater.3c02866.

(7) Sánchez-Iglesias, A.; Winckelmans, N.; Altantzis, T.; Bals, S.; Grzelczak, M.; Liz-Marzán, L. M. High-Yield Seeded Growth of Monodisperse Pentatwinned Gold Nanoparticles through Thermally Induced Seed Twinning. *J. Am. Chem. Soc.* **2017**, *139*, 107–110. https://doi.org/10.1021/jacs.6b12143.

(8) Mushtaq, F.; Raza, Z. A.; Batool, S. R.; Zahid, M.; Onder, O. C.; Rafique, A.; Nazeer, M. A. Preparation, Properties, and Applications of Gelatin-Based Hydrogels (GHs) in the Environmental, Technological, and Biomedical Sectors. *Int. J. Biol. Macromolecules* **2022**, *218*, 601–633. https://doi.org/10.1016/j.ijbiomac.2022.07.168.

(9) Goia, D.; Matijević, E. Tailoring the Particle Size of Monodispersed Colloidal Gold. *Coll. Surf. A* **1999**, *146*, 139–152.
